# Supplementary material for: The Effect of Stress Hormones, Ultraviolet C, and Stilbene Precursors on Expression of Calcineurin B-like Protein (CBL) and CBL-Interacting Protein Kinase (CIPK) Genes in Cell Cultures and Leaves of Vitis amurensis Rupr
Source: Plants (Basel). 2023 Apr 5;12(7):1562. doi: 10.3390/plants12071562 (PMC10147091; doi:10.3390/plants12071562)
Supplement: Supplementary file 1 [file plants-12-01562-s001.zip › plants-2320610-supplementary.pdf]

Table S1. Individual stilbene content (mg/g of dry weight, DW) in the *Vitis amurensis* cell culture V7 and leaves in control conditions (V7, L) and after salicylic acid (SA 50, SA 200 µM), methyl jasmonate acid (MeJa 50, MeJa 200 µM), *p*-coumaric acids acid (CA 100, CA 500 µM) feeding and after ultraviolet C treatment (UV-C 1 h, UV-C 24 h).
* – $p < 0.05$; ** – $p < 0.01$ versus values of total stilbene levels in the V7 cell line (V7, for cell culture samples) or in grape leaves (L, for leaf samples) cultivated in control conditions.

Treatments	(1)[a] *t*-resveratrol diglucoside, mg/g DW	(2) *t*-piceid, mg/g DW	(3) *cis*-piceid, mg/g DW	(4) *t*-piceatannol, mg/g DW	(5) *t*-resveratrol, mg/g DW	(6) *cis*-resveratrol, mg/g DW	(7) ε-viniferin, mg/g DW	(8) δ-viniferin, mg/g DW
V7	**0.23 ±0.05**	**0.04 ±0.01**	**0.01 ±0.01**	**0**	**0.03 ±0.02**	**0**	**0.01 ±0.01**	**0.05 ±0.02**
V7 with CA 100 µM	0.68 ±0.18*	0.13 ±0.03*	0.02 ±0.01	0.01 ±0.01	0.14 ±0.04*	0.01 ±0.01	0.10 ±0.04*	0.27 ±0.08*
V7 with CA 500 µM	0.75 ±0.04**	0.14 ±0.03*	0.01 ±0.01	0.01 ±0.01	0.21 ±0.06**	0	0.09 ±0.03*	0.24 ±0.05*
V7 with MeJa 50 µM	0.72±0.12**	0.25 ±0.11*	0.01 ±0.01	0	0.12 ±0.03*	0	0.05 ±0.02	0.14 ±0.04
V7 with MeJa 200 µM	1.19 ±0.21**	0.13 ±0.02*	0.01 ±0.01	0.01 ±0.01	0.09 ±0.03	0.01 ±0.01	0.35 ±0.20*	0.60 ±0.32**
V7 with SA 50 µM	0.73 ±0.10**	0.10 ±0.03	0	0	0.24 ±0.10**	0	0.06 ±0.02	0.18 ±0.05*
V7 with SA 200 µM	0.77 ±0.08**	0.11 ±0.04	0	0.01 ±0.01	0.14 ±0.02*	0	0.09 ±0.02*	0.19 ±0.05*
V7 after 1 h of UV-C	0.69 ±0.18**	0.09 ±0.04	0.01 ±0.01	0.01 ±0.01	0.14 ±0.05*	0.01 ±0.01	0.11 ±0.02*	0.26 ±0.06*
V7 after 24 h of UV-C	1.31 ±0.45**	0.10 ±0.03	0.03 ±0.01	0.01 ±0.01	0.15 ±0.06*	0.02 ±0.01	0.52 ±0.25**	1.18 ±0.41**
L	**0**	**0.04 ±0.02**	**0**	**0**	**0.01 ±0.01**	**0**	**0.01 ±0.01**	**0.01 ±0.01**
L with CA 100 µM	0	0.16 ±0.08*	0.08 ±0.05*	0.01 ±0.01	0.06 ±0.03*	0	0.01 ±0.01	0.01 ±0.01
L with CA 500 µM	0	0.10 ±0.08	0.04 ±0.03	0.02 ±0.01	0.01 ±0.01	0	0.01 ±0.01	0.01 ±0.01
L with MeJa 50 µM	0	0.06 ±0.02	0.01 ±0.01	0.01 ±0.01	0.05 ±0.02*	0.01 ±0.01	0.01 ±0.01	0.01 ±0.01
L with MeJa 200 µM	0	0.14 ±0.05*	0	0.02 ±0.01	0.02 ±0.01	0.01 ±0.01	0.01 ±0.01	0.01 ±0.01
L with SA 50 µM	0	0.09 ±0.06	0.01 ±0.01	0.01 ±0.01	0.02 ±0.01	0.01 ±0.01	0.01 ±0.01	0.01 ±0.01
L with SA 200 µM	0	0.10 ±0.05	0.03 ±0.02	0	0.12 ±0.06**	0.01 ±0.01	0.01 ±0.01	0.01 ±0.01
L after 1 h of UV-C	0	0.09 ±0.05	0.01 ±0.01	0.01 ±0.01	0.07 ±0.02*	0.05 ±0.02*	0.04 ±0.03	0.01 ±0.01
L after 24 h of UV-C	0	0.14 ±0.04*	0.01 ±0.01	0.01 ±0.01	0.08 ±0.02*	0.09 ±0.02*	0.04 ±0.02	0.01 ±0.01

[a] - the numbers of stilbene compounds in parentheses correspond to the numbers of stilbenes presented on the HPLC chromatogram (Figure 1).

Table S2. Individual calcineurin B-like proteins (*CBL*) and CBL-interacting protein kinases (*CIPK*) genes and their known transcripts in grapevine (Xi et al. 2017).

Gene name	Gene transcript name	Gene ID*	Primers for qPCR	
CBL4	*CBL4-1*	VIT_216s0098g01870.1	5'TGAAGGAGATGGTGCTGGC<br>5'GTTGAAGACTCAGAAATATAG	
	CBL4-2	VIT_216s0098g01870.8	5'TGAAGGAGATGGTGCTGGC<br>5'GTATCAAAGAATCCATCCCTGA	
CBL5	*CBL5*	VIT_202s0025g01630	5'GGAGATGGAAAGATTGATCC<br>5'GAGATCAGACCCTGAAGATTAA	
CBL8	*CBL8*	VIT_202s0025g01640	5'GTAGCCCGGAATCCATCAAT<br>5'GATTCAGATATTGTAGCCTAA	
CBL10a	*CBL10a-1*	VIT_204s0008g00950.1	5'ATTTATCGAGCGCGAGGAAGT<br>5'CAATACTGAGGTTGAAGAATGA	
	CBL10a-2	VIT_204s0008g00950.2	5'ATTTATCGAGCGCGAGGAAGT<br>5'TGACTCTTCCTTATTTGAAGTAA	
	CBL10a-3	VIT_204s0008g00950.3	5'ATTTATCGAGCGCGAGGAAGT<br>5'TGACAAGGTGTTGTTTAAATTATAA	
	CBL10a-4	VIT_204s0008g00950.4	5'ATTTATCGAGCGCGAGGAAGT<br>5'TCGGTTGTTGCCAAAAACTAG	
CBL10b	*CBL10b-1*	VIT_204s0008g00960.1	5'ATGAACTCTACGGAGAGTTC<br>5'AGTATTCACCGCACCCACC	
CBL11	*CBL11*	VIT_219s0015g01070	5'CTACGACACCCATCCCTTTTG<br>5'CTCAAGTTGACGACACCTGA	
CBL12	*CBL12*	VIT_200s1569g00020	5'CATCACCACTACATTCCCAAGT<br>5'CTCACAAGTGGATGACACTTGA	
CBL13	*CBL13-1,2*	VIT_202s0236g00140.1	5'GACGTAAATCAGGATGGGAG<br>5'GTGGATGAGATTGCTACATAA	
	CBL13-3	VIT_202s0236g00140.3	5'GACGTAAATCAGGATGGGAG<br>5'GCTTTTTTTGACAGTTGAGTAA	
CIPK3	*CIPK3*	VIT_206s0009g01840	5'GGAAACTGCTATGCCTTTGGGG<br>5'GTCTGGTGTGGCATCAACTTGA	
CIPK9	*CIPK9-1*	VIT_215s0048g02740.3	5'TGTCTAAAATTGAGGAAACTGCA<br>5'TACTGAAGAATTGAGGT	
	CIPK9-1	VIT_215s0048g02740.4	5'TGTCTAAAATTGAGGAAACTGCA<br>5'GGAGTTCCACAAGGCATGCTAA	
CIPK12	*CIPK12*	VIT_210s0003g01420	5'CCCGTGTTGCAGAACCTTG<br>5'TTTGCCCTCAGATACTGAATAG	

CIPK21	*CIPK21-1*	VIT_211s0052g01700.2	5'AGGTGGCTCCCACTCATTGT<br>5'CAATGGCTATTCTTCCTCTTGA	
	CIPK21-2	VIT_211s0052g01700.3	5'AGGTGGCTCCCACTCATTGT<br>5'ATATTCAGGAAACTGAATGGTAA	
CIPK24	*CIPK24*	VIT_211s0016g05420	5'GAGGTTTTTGAAGTTGCACCAT<br>5'CTAAGAACAATGACTTGCTGA	
CIPK27	*CIPK27*	VIT_208s0058g01090	5'CGCCTTGATAATGGAGATATATC<br>5'GAGCCGAGCACCGAGAGCTGA	
CIPK28	*CIPK28*	VIT_206s0004g07870	5'GACCAAATGGTAATTTCATAATCG<br>5'ACCCAAGTCGCCGGCGATTAA	
CIPK29	*CIPK29*	VIT_205s0020g04570	5'CTGTTGGTCGAAGTTTCAGAAG<br>5'TGGGAGGGTAGTGGGATGTAG	
CIPK30	*CIPK30*	VIT_209s0070g00160	5'GCGACGTGGTATTGAAGCTA<br>5'CCAAAATTCGGCAGTTGCTTGA	
CIPK31	*CIPK31*	VIT_211s0016g00200	5'GGGAAATTAGCGATAGGAGC<br>5'TGGACGTCCACCCCAGCCTGA	
CIPK32	*CIPK32*	VIT_204s0008g05770	5'GGCCGGAAAGGGAGGTTAT<br>5'ATATTGTTGAATGTGAAAATTAA	
CIPK33	*CIPK33*	VIT_209s0070g00140	5'CTGGAGTACACCAAGTTTTGTG<br>5'TATGACATGTGAGGAATGGTAG	
CIPK34	*CIPK34*	VIT_208s0058g01040	5'GTGAAGAAGTCCAGTGGGGA<br>5'CATCTCACCTCCAGCTTCATAA	
CIPK35	*CIPK35*	VIT_216s0022g00350	5'GGTTATGGCCAGCAAAATGAAG<br>5'AGGTGTTTATCATCGGGATTGA	
CIPK36	*CIPK36*	VIT_210s0003g01410	5'GGTTAAGAAAAAGGATGGGACG<br>5'GCAACAGCCACAACCACAATAG	
CIPK37	*CIPK37*	VIT_213s0067g02480	5'GGAAATGAAGAAGTCCAGTGG<br>5'ACTGCAGGTTCATTCTTCTTGA	
CIPK38	*CIPK38*	VIT_206s0004g07830	5'GAAATTTGAGGGCCTGAAGG<br>5'GCAACAACAACAGCTTTCCTGA	
CIPK39	*CIPK39*	VIT_210s0003g04020	5'GGAAACTGCTATGCCTTTGGGG<br>5'GTCTGGTGTGGCATCAACTTGA	
CIPK40	*CIPK40*	VIT_205s0020g00830	5'GTGGCAGCAGAGGTGTTTG<br>5'GCCAAGAGCTATTGAAAGTTTGA	
CIPK41	*CIPK41-1*	VIT_218s0001g07980.1	5'GATGTAAACGCTGTGTTTGATG<br>5'GACGAAGAGTAAAAAGGGTTGA	
	CIPK41-2	VIT_218s0001g07980.2	5'GATGTAAACGCTGTGTTTGATG<br>5'AAAATATCAGATCTGACCAATGA	
	CIPK41-3	VIT_218s0001g07980.3	5'GATGTAAACGCTGTGTTTGATG<br>5'GTTCTCTTCACAACATATCTGA	

	CIPK41-4	VIT_218s0001g07980.4	5'GATGTAAACGCTGTGTTTGATG<br>5'CTGGTTTAGCAATCCTAACTAA	
	CIPK41-5	VIT_218s0001g07980.5	5'GATGTAAACGCTGTGTTTGATG<br>5'GAGGTTGGAACAACATGCTAA	
	CIPK41-6	VIT_218s0001g07980.6	5'GATGTAAACGCTGTGTTTGATG<br>5'TTGCGTTTTATTGCCACTGTGA	

*ORCAE platform, online resource for community annotation of eukaryotes (http://bioinformatics.psb.ugent.be/orcae/overview/Vitvi).

Figure S1. Nucleotide sequence of the *VvCBL* and *VaCIPK* transcripts.

>**VvCBL4-1**,V.vinifera v2.1|VIT_216s0098g01870.1
ATGGGCTGCTTTTGCTCTAAGAAAGCTAGACAAACACCAGGATACGAGGAGCCTACTGTTCTTGCTGCTGAAACACCTTTTACTGTGTGCGAAGTAGAAGCCTTGTATGAGCTATTTAAGAAATTAAGCAGTT
CCATCATAGATGACGGGCTTATCCACAAGGAAGAATTCCAGCTTGCACTCTTCAGGAACAGAAATAAGAGGAATCTCTTTGCAGATAGGATATTTGACCTGTTTGATGTCAAGCGAAATGGGGTTATTGAGTT
TGGAGAATTTGTTCGTTCATTGGGTGTGTTTCACCCAAATGCACCTGAAGCAGACAAAATTTCTTTTGCTTTTCGACTGTATGATCTAAGGCACACCGGGTATATTGAGCGAGAGGAGTTGAAGGAGATGGTG
CTGGCGCTTCTTCATGAATCAGATCTGATACTTTCAGATGAATATGTTGAACAAATTGTGGATAAGACTTTCAATGATGCAGATTCAAATGGTGATGGAAGGATTGACCCAGAAGAGTGGAAGGAATTTGTAT
CAAAGAATCCATCCCTGATAAAGAACATGACTCTTCCATACCTAAAGGACATAACATTGGCATTTCCCAGCTTTGTGATAAGCTCTGAAGTTGAAGACTCAGAAATATAG
>**VvCBL4-2,**V.vinifera v2.1|VIT_216s0098g01870.8
ATGGGCTGCTTTTGCTCTAAGAAAGCTAGACAAACACCAGGATACGAGGAGCCTACTGTTCTTGCTGCTGAAACACCTTTTACTGTGTGCGAAGTAGAAGCCTTGTATGAGCTATTTAAGAAATTAAGCAGTT
CCATCATAGATGACGGGCTTATCCACAAGGAAGAATTCCAGCTTGCACTCTTCAGGAACAGAAATAAGAGGAATCTCTTTGCAGATAGGATATTTGACCTGTTTGATGTCAAGCGAAATGGGGTTATTGAGTT
TGGAGAATTTGTTCGTTCATTGGGTGTGTTTCACCCAAATGCACCTGAAGCAGACAAAATTTCTTTTGCTTTTCGACTGTATGATCTAAGGCACACCGGGTATATTGAGCGAGAGGAGTTGAAGGAGATGGTG
CTGGCGCTTCTTCATGAATCAGATCTGATACTTTCAGATGAATATGTTGAACAAATTGTGGATAAGGTGCAGATTACTGTAACTTGCCACCTGCATTTTCATGCTTATTTGATCTATATATTCAAAATTTCTTGT
CTTCTCCACAGACTTTCAATGATGCAGATTCAAATGGTGATGGAAGGATTGACCCAGAAGAGTGGAAGGAATTTGTATCAAAGAATCCATCCCTGA
>**VvCBL5**,V.vinifera v2.1|VIT_202s0025g01630.1
ATGGGCTGTGCTTGTATGAAGCAGCGGCAGGAACGCAAAAGCGCAGTGCTTCTTGCTGCTCAAACTTGCTTTAAAGTGGCAGATGTGGAAGCTTTGTATGAGCTGTTCAGGAAATTAAGCAGTTCTGTGGTTG
ATGATGGACTTATCAGCAAAGATGAGTTTCGGCTTGGCTTATTCAGGAACAGCAAGAAGCAGAGTCTTCTTGCAGATAGGATCTTCAATCTGTTCGACTTGAAACATGATGGGATGATAGAGTTTGGAGAATT
CATCCGGTCTTTGAGCATTTTCCACCCAGATGCTCCCCAAGAAGAAAAAATTGCTTTTGCATTTCAATTATACAATACAGGACAGACAGGCTTCATCGAACATGAGGAGGTCAGGGAGATGGTTTTCGCCTTC
CTGAATGAGTCAGACTTGATCCTTTCTGATGACCTTGTTGAAGCCATTATTGATAAGACTTTCAGTGAAGTAGATACAAAAGGAGATGGAAAGATTGATCCAGAAGAGTGGAATGAATTCATTTCTCGGAATC
CATCCATACTAAAGAACATGACCATCCCATATTTGAAGGATATCGGAACTTCATTTCCCAGTTTTGTGCTGAGATCAGACCCTGAAGATTAA
>**VvCBL8**,V.vinifera v2.1|VIT_202s0025g01640.1
ATGCTTGCTTTTACACGTTGCTTTTGCTTGAAGAAGGCTAAGCAGCATCCTGGGTACGAGGACCCCACTACTCTTGCTTCTGAAACACCTTTTACTGTAAATGAGGTCGAGGCATTATACGATCTATTCAAGAA
ACTAAGCAGTTCCATAATTGATGATGGTTTCATACACAAGGAAGAATTTCAGTTTGCTCTTTTCAGGAACAGAAGTAAGCAGAGTCTCTTTGCAGACAGGGTGTTTGATCTTTTTGATATTAAGCATAATGGG
GTCATTGAATTTGGAGAATTTGTTCGATCATTAAGCATTTTTCACCCAAATGCTCCCAACGAAGACAAAATGGAATTTGCATTTAAATTGTATGATCTAAGGCAGACTGGCTACATTGAGCGTGAAGAGTTGA
CGGAGATGGTTTCAGCTATTCTTAATGAATCGGATCTGGAAATTTCAGAAGACATTGTTGAATCAATCGTGGATAAGACAATGGCAGAGGCAGATTTGAAAGGAGATGGGAAGATTGATAAAGAAGAGTGG
AAGGAATTTGTAGCCCGGAATCCATCAATCCTAAGAAACATGACTCTTCCATACTTAAAGCAGATAACTCTAGCATTTCCCAGCTTTGTGCTGAACTCTGAGGTACAAGATTCAGATATTGTAGCCTAA
>**VvCBL10a1**,V.vinifera v2.1|VIT_204s0008g00950.1
ATGAGTTCTTGGCAGGGAACGGCGAACTCTACATACAATTATTTGAGATCGAGCTCTCTCACAATAGGAGAGAAGTTATGTGCTGCTTTTATACCGTTTATAGGCATAATTGAAGCTCTGGTATTCGCTGTGGC
CGGTTGCTGCGACTTCCACAAAAACAAGCATCACAAGAAACTGAGATACGGGTTCAGAGAGCTCTCGCAACTCGCCCGTGAATCGCAATTTACTGTCAATGAAGTGGAGGCACTGTATGAGTTGTTTAAGAA
GTTAAGTAGCTCAATTATTGACGATGGCTTGATTCACAAGGAAGAGCTTCAATTGGCATTGTTCAAGTCTCCGCATAGTGAGAATCTTTTCCTAGACAGGGTTTTTTATCTTTTTGATGAAAAGAAAAATGGTG
TGATTGAATTTGACGAATTTGTCCATGCACTCAATGTCTTCCATCCCTATGCTCCTACAGAAGACAAAATAGATTTTGCATTTAGGTTGTATGATTTGAGACAAACTGGATTTATCGAGCGCGAGGAAGTTAAG
CAAATGGTTATTGCCATTTTGTTGGAATCTGATATGAATCTATCAGATGATCTTCTTGAGGCTATCATTGACAAGACATTTGCTGATGCTGATGCTGACAATGATGGTAAAATCAACAAAGAAGAATGGAAAG
CTTTTGTACTTCGACACCCGAACCTCTTGAAGAACATGACTCTTCCTTATTTGAAGGACATCACCACAGTATTTCCAAGCTTTGTTTTCAATACTGAGGTTGAAGAATGA
>**VvCBL10a2**,V.vinifera v2.1|VIT_204s0008g00950.2
ATGAGTTCTTGGCAGGGAACGGCGAACTCTACATACAATTATTTGAGATCGAGCTCTCTCACAATAGGAGAGAAGTTATGTGCTGCTTTTATACCGTTTATAGGCATAATTGAAGCTCTGGTATTCGCTGTGGC
CGGTTGCTGCGACTTCCACAAAAACAAGCATCACAAGAAACTGAGATACGGGTTCAGAGAGCTCTCGCAACTCGCCCGTGAATCGCAATTTACTGTCAATGAAGTGGAGGCACTGTATGAGTTGTTTAAGAA
GTTAAGTAGCTCAATTATTGACGATGGCTTGATTCACAAGGAAGAGCTTCAATTGGCATTGTTCAAGTCTCCGCATAGTGAGAATCTTTTCCTAGACAGGGTTTTTTATCTTTTTGATGAAAAGAAAAATGGTG
TGATTGAATTTGACGAATTTGTCCATGCACTCAATGTCTTCCATCCCTATGCTCCTACAGAAGACAAAATAGATTTTGCATTTAGGTTGTATGATTTGAGACAAACTGGATTTATCGAGCGCGAGGAAGTTAAG
CAAATGGTTATTGCCATTTTGTTGGAATCTGATATGAATCTATCAGATGATCTTCTTGAGGCTATCATTGACAAGACATTTGCTGATGCTGATGCTGACAATGATGGTAAAATCAACAAAGAAGAATGGAAAG

CTTTTGTACTTCGACACCCGAACCTCTTGAAGAACATGACTCTTCCTTATTTGAAGTAA
>**VvCBL10a3**,V.vinifera v2.1|VIT_204s0008g00950.3
ATGAGTTCTTGGCAGGGAACGGCGAACTCTACATACAATTATTTGAGATCGAGCTCTCTCACAATAGGAGAGAAGTTATGTGCTGCTTTTATACCGTTTATAGGCATAATTGAAGCTCTGGTATTCGCTGTGGC
CGGTTGCTGCGACTTCCACAAAAACAAGCATCACAAGAAACTGAGATACGGGTTCAGAGAGCTCTCGCAACTCGCCCGTGAATCGCAATTTACTGTCAATGAAGTGGAGGCACTGTATGAGTTGTTTAAGAA
GTTAAGTAGCTCAATTATTGACGATGGCTTGATTCACAAGGAAGAGCTTCAATTGGCATTGTTCAAGTCTCCGCATAGTGAGAATCTTTTCCTAGACAGGGTTTTTATCTTTTTGATGAAAAGAAAAATGGTG
TGATTGAATTTGACGAATTTGTCCATGCACTCAATGTCTTCCATCCCTATGCTCCTACAGAAGACAAAATAGATTTTGCATTTAGGTTGTATGATTTGAGACAAACTGGATTTATCGAGCGCGAGGAAGTTAAG
CAAATGGTTATTGCCATTTTGTTGGAATCTGATATGAATCTATCAGATGATCTTCTTGAGGCTATCATTGACAAGGTGTTGTTTAAATTATAA
>**VvCBL10a4**,V.vinifera v2.1|VIT_204s0008g00950.4
ATGAGTTCTTGGCAGGGAACGGCGAACTCTACATACAATTATTTGAGATCGAGCTCTCTCACAATAGGAGAGAAGTTATGTGCTGCTTTTATACCGTTTATAGGCATAATTGAAGCTCTGGTATTCGCTGTGGC
CGGTTGCTGCGACTTCCACAAAAACAAGCATCACAAGAAACTGAGATACGGGTTCAGAGAGCTCTCGCAACTCGCCCGTGAATCGCAATTTACTGTCAATGAAGTGGAGGCACTGTATGAGTTGTTTAAGAA
GTTAAGTAGCTCAATTATTGACGATGGCTTGATTCACAAGGAAGAGCTTCAATTGGCATTGTTCAAGTCTCCGCATAGTGAGAATCTTTTCCTAGACAGGGTTTTTATCTTTTTGATGAAAAGAAAAATGGTG
TGATTGAATTTGACGAATTTGTCCATGCACTCAATGTCTTCCATCCCTATGCTCCTACAGAAGACAAAATAGATTTTGCATTTAGGTTGTATGATTTGAGACAAACTGGATTTATCGAGCGCGAGGAAGTGAGT
AGACATTTTTTTTTCGTATCGGTTGTTGCCAAAAACTAG
>**VvCBl10b1**.V.vinifera v2.1|VIT_204s0008g00960.1
ATGAACTCTACGGAGAGTTCTTGGAGATCGAGCCCTGTAACAATGGGTGGAGGTTTTGTGCTTCTTTCATACCATTCTTCTCCATAATTGAAGGTCTGGTGTTCGCTGTAGCCAATTTCTGCAGTATTCACCG
CACCCACCACCACAGGAATTTACGGTACGGGCTCAGAGAGCTGACGCAGCTCGCCGGTGAAACGCAATTTACTGTTAATGAAGTGGAGGCTTTGTATGAGTTGTTTAAGAAGTTAAGTAGCTCAATTATTGAT
GATGGCTTGATTCACAAGGAAGAGCTCCAATTAGCACTTCTCAAGTCTCCATGTGGCCAGAACCTTTTTTTAGACAGGGTATTTTATCTTTTTGATGAAAGGAAAAATGGTGCAATTGAGTTTGATGAATTTGT
CCGTGCACTCAGTGTCTTCCATCCCTATGCCCCAATGGAAGACAAAATAGATTTTGCATTTAGGCTGTATGACCTGAGACAAACCGGATTTATTGAGAGAGAAGATGTTAAGCAAATGGTAATTGTCACTTTG
ATGGAATCGGAGATGGATCTGTCAGATGATCTTCTTGAGGCTATCATTGACAAGACATTTGTTGATGCTGATGCTGACAGGGATGGCAAAATCAGTAAAGAAGAATGGAGGGCTTTTGTACTTAGACACCCA
AATCTCTTAAACAACATGAGTCTTTCTTATTTGAAGGATGTCACTACAGTATTTCCGAGTTTTGTCATCAATTCTGAGGTTGAGGACTGA
>**VvCBl10b2.**V.vinifera v2.1|VIT_204s0008g00960.2
ATGGTTGGGATATGTTGTTTTCGACAGAGATCGAGCCCTGTAACAATGGGTGGAGGTTTTGTGCTTCTTTCATACCATTCTTCTCCATAATTGAAGGTCTGGTGTTCGCTGTAGCCAATTTCTGCAGTATTCAC
CGCACCCACCACCACAGGAATTTACGGTACGGGCTCAGAGAGCTGACGCAGCTCGCCGGTGAAACGCAATTTACTGTTAATGAAGTGGAGGCTTTGTATGAGTTGTTTAAGAAGTTAAGTAGCTCAATTATTG
ATGATGGCTTGATTCACAAGGAAGAGCTCCAATTAGCACTTCTCAAGTCTCCATGTGGCCAGAACCTTTTTTTAGACAGGGTATTTTATCTTTTTGATGAAAGGAAAAATGGTGCAATTGAGTTTGATGAATTT
GTCCGTGCACTCAGTGTCTTCCATCCCTATGCCCCAATGGAAGACAAAATAGATTTTGCATTTAGGCTGTATGACCTGAGACAAACCGGATTTATTGAGAGAGAAGATGTTAAGCAAATGGTAATTGTCACTT
TGATGGAATCGGAGATGGATCTGTCAGATGATCTTCTTGAGGCTATCATTGACAAGACATTTGTTGATGCTGATGCTGACAGGGATGGCAAAATCAGTAAAGAAGAATGGAGGGCTTTTGTACTTAGACACC
CAAATCTCTTAAACAACATGAGTCTTTCTTATTTGAAGGATGTCACTACAGTATTTCCGAGTTTTGTCATCAATTCTGAGGTTGAGGACTGA
>**VvCBL11**,V.vinifera v2.1|VIT_219s0015g01070.1
ATGTTGCAGTGCTTAGAGGGATTTAAGCATTTAATTGCTTCCCTACTGCGGTGTTGTGATCTTGATTTGTATAATCAAGCAAGAGGTCTTCAAGATCCTGAAGTTCTTGCAAGGGAAACAGTGTTTAGTGTAAG
TGAAATCGAGGCACTATACGAGCTGTTTAAGAAGATCAGCAGCGCTGTGATTGATGACGGTCTGATCAACAAGGAAGAGTTTCAATTGGCTTTGTTCAAAACAAACAAAAAGGAAAGCTTATTTGCTGATCG
GGTATTTGACTTGTTTGACACAAAGCATAACGGAATACTTGGCTTTGAGGAGTTTGCTCGTGCTCTATCTGTTTTTCACCCCAACGCACCTATCGACGACAAGATTGAGTTTTCTTTTCAGTTATACGATCTCAA
GCAGCAGGGTTTCATTGAGAGGCAGGAGGTGAAGCAAATGGTAGTGGCCACACTTGCTGAATCTGGTATGAATCTTTCGGATGATGTTATAGAAAGTATCATTGACAAGACCTTTGAGGAAGCTGACACAAA
ACATGATGGAAAGATTGACAAAGAAGAGTGGAGAAGCCTTGTTCTACGACACCCATCCCTTTTGAAGAATATGACTCTTCAATACCTCAAGGACATCACCACCACATTCCCAAGCTTTGTCTTTCATTCTCAA
GTTGACGACACCTGA
>**VvCBL12.**V.vinifera v2.1|VIT_200s1569g00020.1
ATGGTAGTGGCTACGCTTGCTGAATCTGGCATGAATCTTTCAGATGATGTAATAGAAAGTATCATTGATAAGACCTTTGAGGAAGCTGATACTAAACATGATGGGAAGATAGACAAGGAAGAATGGCGAAGC
CTTGTTTTGCGACATCCATCTCTTTTGAAGAATATGACCCTTCAATACCTAAAAGACATCACCACTACATTCCCAAGTTTTGTGTTTCACTCACAAGTGGATGACACTTGA
>**VvCBL13-1,2**.V.vinifera v2.1|VIT_202s0236g00140.1
ATGGGCTGCTTTCAGTCAACAGCAAGAAAGCAGTTTCCTGGACATGAGGACCCAGTAATTCTTGCTTCCCAAACAGCTTTTAGTGTGAGTGAAGTTGAAGCACTATTTGAGCTATTCAAGAGCATTAGCAGTT
CTGTAATTGATGATGGGTTAATAAACAAGGAAGAGTTTCAATTGGCTCTATTCAAGAACAGAAAGAAGGAGAATCTCTTTGCAAATAGGATCTTTGATCTTTTTGATGTTAAGCGGAAGGGAGTCATTGATTT
TGGTGACTTTGTCAGGTCACTAAATGTTTTCCATCCAAATGCCCCACAAGAAGACAAAATTGATTTTTCATTCAAGCTCTATGATTTGGATAGCACAGGATTCATTGAGCGGCAAGAGGTCAAACAAATGTTA
ATTGCACTTCTGTGTGAATCTGAAATGAAGTTGGCTGATGAAACCATCGAGATAATACTTGATAAGACATTCTTGGAAGCTGACGTAAATCAGGATGGGAGGATTGATAAATCTGAGTGGCAGAATTTTGTTT
CTCGGAACCCATCATTGTTGAAGATAATGACACTTCCTTATCTAAGGGACATAACGACAACCTTTCCGAGCTTTGTGTTTAATTCAGAGGTGGATGAGATTGCTACATAA
>**VvCBL13-3**.V.vinifera v2.1|VIT_202s0236g00140.3
ATGGGCTGCTTTCAGTCAACAGCAAGAAAGCAGTTTCCTGGACATGAGGACCCAGTAATTCTTGCTTCCCAAACAGCTTTTAGTGTGAGTGAAGTTGAAGCACTATTTGAGCTATTCAAGAGCATTAGCAGTT
CTGTAATTGATGATGGGTTAATAAACAAGGAAGAGTTTCAATTGGCTCTATTCAAGAACAGAAAGAAGGAGAATCTCTTTGCAAATAGGATCTTTGATCTTTTTGATGTTAAGCGGAAGGGAGTCATTGATTT
TGGTGACTTTGTCAGGTCACTAAATGTTTTCCATCCAAATGCCCCACAAGAAGACAAAATTGATTTTTCATTCAAGCTCTATGATTTGGATAGCACAGGATTCATTGAGCGGCAAGAGGTCAAACAAATGTTA
ATTGCACTTCTGTGTGAATCTGAAATGAAGTTGGCTGATGAAACCATCGAGATAATACTTGATAAGACATTCTTGGAAGCTGACGTAAATCAGGATGGGAGGATTGATAAATCTGAGTGGCAGAATTTTGTTT
CTCGGAACCCATCATTGTTGAAGATAATGACACTTCCTTATCTAAGGTGGGTAATCACAATGTTCTGGTCTCAAGCATCAACAATCATCACAATGAAGCTTTTTTTGACAGTTGAGTAA

>**VvCIPK3**.V.vinifera v2.1|VIT_206s0009g01840.1

ATGAATCCACCTAAAGTCAAGCGTCGAGTTGGGAAATATGAGGTGGGAAGGACAATTGGGGAGGGTACATTTGCGAAAGTCAAGTTTGCAAGGAATTCTGAGACTGGGGAACCGGTTGCTATAAAGATCCT
GGACAAAGAGAAGGTTCTGAAACACAAAATGGCTGAACAGATAAAGCGGGAAGTAGCAACAATGAAACTAATAAAGCATCCAAATGTTGTTCGCTTATATGAGGTAATGGGGAGCAAGACAAAGATATTC
ATTGTTTTAGAGTTTGTTACTGGGGGAGAGCTCTTTGACAAAATTGTAAACCATGGGCGAATGAGGGAGGATGAAGCACGAAGGTATTTCCAGCAGCTTATTAATGCTGTTGATTATTGTCACAGTAGGGGTG
TCTACCACAGAGACTTAAAGCCAGAAAATCTGCTGTTGGATGCTTATGGAAACCTCAAAGTTTCTGATTTTGGATTAAGTGCATTGTCCCAACAAGTCAGGGATGATGGCCTGCTTCACACTACCTGTGGAAC
TCCAAACTATGTTGCCCCTGAGGTCCTTAATGACAGAGGCTATGATGGTGCAACTGCAGACTTGTGGTCCTGTGGTGTTATACTCTTTGTATTGCTTGCAGGGTACTTGCCTTTTGATGATTCTAATCTTATGAT
CCTATATAAAAAAATATCAGCTGCAGAATTTACTTGTCCACCTTGGCTCTCTTTCGGTGCTATGAAATTGATAACTCGCATTTTGGATCCCAACCCTATGACTCGTATTACAATCCCTGAAATTCTAGAAGATG
AATGGTTTAAGAAAGGTTATAAGCCCCCTGTGTTTGAGGAGAAAGGAGATGCAAACTTGGATGATGTAGAAGCTGTATTTAAAGATTCAGAAGAACATCATGTTACAGAGAAAACGGAGGAACAACCAGCA
GCAATGAATGCATTTGAATTAATTTCCATGTCAAGAGGGCTCAACCTTGGGAATTTGTTTGATATAGAACAGGGATTCAAGAGAGAAACGAGGTTCACGTCAAAATGCCCTGCCAATGAGATAATTCATAAG
ATTGAAGAAGCTGTGAAGCCTCTTGGGTTTGATGTTCACAAGAAAAACTATAAGATGAGGCTTGAAAATATGAAAGCTGGGAGAAAAGGGAACCTTAATGTGGCTACAGAGGTATTTCAAGTGGCACCTTCT
CTTCATATGGTTGAGGTTCGAAAAGCAAAGGGAGATACTCTGGAGTTCCATAAGTTCTACAAAAATCTTTCGACCTGCCTTGAGGATGTGGTTTGGAAAACTGAGGATGACATGCAAGAAACGAAATAA
>**VvCIPK9-1**.V.vinifera v2.1|VIT_215s0048g02740.3
ATGAGCATGAAGGTTCCGGCGACGAGGACGCGTGTCGGGAGATACGAAATCGGTAAAACCTTGGGTGAGGGGAGCTTCGCCAAGGTCAAGTACGCTCGCAACTCCGACACCGGCGATAGCGTCGCCATCAA
GGTGCTCGACCGCGATCATGTTCTCCGGCACAAGATGGTGGAACAGATAAAAAGAGAGATATCGACAATGAAGCTGATTAAGCATCCGAATGTTGTGACGATCTTCGAGGTTATGGCGAGCAAAACAAAGA
TCTACATTGTCGTTGAGTTTGTTGACGGAGGAGAGCTCTTTGACAGAATTGCCAAGAATGGGAGACTTAAAGAGGATGAAGCCAGGAGATACTTCCAGCAGCTCATTAATGCTGTAGATTATTGCCACAGTA
GAGGCGTGTACCATAGAGATTTGAAGCCCGAGAATTTACTTCTGGATTCATACGGTGTTCTTAAAATATCAGACTTTGGATTGAGTGCCCTTTCACAGCAAGTGAGGGAAGATGGGCTGTTTCACACTGCCTG
TGGGACGCCAAATTATGTTGCTCCTGAGGTTCTCAATAATAAAGGATATGATGGTGCATCAGCTGATATATGGTCTTGTGGGGTCATTCTATTTGTACTTCTGGCTGGGTACTTGCCTTTTGATGAAGTAAGCC
TCATGGCTTTGTATAAAAAGATCTGCAAGGCTGATTTCTCATGTCCATCATGGTTATCACCTGGTGCAAAGAAGCTGATAAACCGTATTCTTGATCCCAATCCTGTAACTCGGATAACAATTCCTGAAATCTTG
GAAGATGGATGGTTCAAGAAAGGGTACCAGCCAACATGTTTTCAGCAGGAAGAGGATGTGAATGTTGATGATGTGGATGCTGCTTTCAATAATTCAGAGGAAAATCTTGTGACAGAAAGGAAAGAAAAACC
TGTCTCCATGAATGCCTTCGAGCTTATTTCTAGGTCTCAGAGTTTCAATCTTGAAAACTTGTTCGGGAAGCAGGCGGGTCTTGTGAAGCGAGAGACAAGTTTCACTTCCCAGCGCCCTGCCCATGAGATTATGT
CTAAAATTGAGGAAACTGCAAAGCCTCTAGGTTTCAACGTTCGCAAGCAAAACTACAAGATGAAGTTGCAAGGTGACAAAACTGGACGGAAGGGTCATCTCTCTGTAGCTACTGAGGTGTTTGAGGTGGCTC
CCTCCTTGCACATGGTGGAGCTTCGGAAAACTGGTGGTGACACACTGGAGTTCCACAAGTTCTACAAAAGCTTCTCTTCAGGATTAAAAGATATAGTCTGGAAATCAGAAGCGAATACTGAAGAATTGAGGT
GA
>**VvCIPK9-2.**V.vinifera v2.1|VIT_215s0048g02740.4
ATGAGCATGAAGGTTCCGGCGACGAGGACGCGTGTCGGGAGATACGAAATCGGTAAAACCTTGGGTGAGGGGAGCTTCGCCAAGGTCAAGTACGCTCGCAACTCCGACACCGGCGATAGCGTCGCCATCAA
GGTGCTCGACCGCGATCATGTTCTCCGGCACAAGATGGTGGAACAGATAAAAAGAGAGATATCGACAATGAAGCTGATTAAGCATCCGAATGTTGTGACGATCTTCGAGGTTATGGCGAGCAAAACAAAGA
TCTACATTGTCGTTGAGTTTGTTGACGGAGGAGAGCTCTTTGACAGAATTGCCAAGAATGGGAGACTTAAAGAGGATGAAGCCAGGAGATACTTCCAGCAGCTCATTAATGCTGTAGATTATTGCCACAGTA
GAGGCGTGTACCATAGAGATTTGAAGCCCGAGAATTTACTTCTGGATTCATACGGTGTTCTTAAAATATCAGACTTTGGATTGAGTGCCCTTTCACAGCAAGTGAGGGAAGATGGGCTGTTTCACACTGCCTG
TGGGACGCCAAATTATGTTGCTCCTGAGGTTCTCAATAATAAAGGATATGATGGTGCATCAGCTGATATATGGTCTTGTGGGGTCATTCTATTTGTACTTCTGGCTGGGTACTTGCCTTTTGATGAAGTAAGCC
TCATGGCTTTGTATAAAAAGATCTGCAAGGCTGATTTCTCATGTCCATCATGGTTATCACCTGGTGCAAAGAAGCTGATAAACCGTATTCTTGATCCCAATCCTGTAACTCGGATAACAATTCCTGAAATCTTG
GAAGATGGATGGTTCAAGAAAGGGTACCAGCCAACATGTTTTCAGCAGGAAGAGGATGTGAATGTTGATGATGTGGATGCTGCTTTCAATAATTCAGAGGAAAATCTTGTGACAGAAAGGAAAGAAAAACC
TGTCTCCATGAATGCCTTCGAGCTTATTTCTAGGTCTCAGAGTTTCAATCTTGAAAACTTGTTCGGGAAGCAGGCGGGTCTTGTGAAGCGAGAGACAAGTTTCACTTCCCAGCGCCCTGCCCATGAGATTATGT
CTAAAATTGAGGAAACTGCAAAGCCTCTAGGTTTCAACGTTCGCAAGCAAAACTACAAGATGAAGTTGCAAGGTGACAAAACTGGACGGAAGGGTCATCTCTCTGTAGCTACTGAGGTGTTTGAGGTGGCTC
CCTCCTTGCACATGGTGGAGCTTCGGAAAACTGGTGGTGACACACTGGAGTTCCACAAGGCATGCTAA
>**VvCIPK12**.V.vinifera v2.1|VIT_210s0003g01420.1
ATGGCAGACAGGCAGAGCAAGGAGAACACAAGCAAAAGGAAGAACGGGGAGAACCAATCTTTGCTTCTGGGGCGGTACGAGATCGGGAAGCTTCTGGGGCATGGGACATTCGCCAAGGTCTACCACGCCA
GGAACGTCGGATCCAACGAGAGCGTGGCCATCAAAGTCATCGACAAGGAGAAGATCCTCAAAGGCGGTTTGATCGCACATATCAAGCGCGAGATCTCCATACTCCGCCGCGTTCGCCATCCCAACATCGTTC
AATTGTTTGAAGTCATGGCCACCAAGGCTAAGATCTACTTCGTCATGGAGTACGTCAGGGGCGGAGAGCTCTTCAATAAGGTCGCCAAAGGCCGCCTCAAGGAGGAGGTCGCTCGCAAGTATTTCCAGCAGT
TGATCTCCGCCGTCGGCTTCTGCCACGCCAGAGGGGTCTACCACCGCGACCTCAAGCCCGAGAATCTCTTACTGGACGAAGCCGGTGACCTCAAAGTCTCCGATTTTGGGCTTAGCGCTGTCTCTGATCAGAT
GAGGCAGGATGGCCTGTTCCACACCTTTTGTGGGACTCCGGCGTATGTGGCACCTGAGGTTTTGGCCCGGAAGGGCTATGACGCATCCAAGGTTGATATATGGTCGTGCGGTGTGATACTCTTCGTCTTGATG
GCTGGGTATCTCCCTTTCCATGATCAGAACATCATGGCCATGTACAAGAAAATCTACAAGGGTGAATTCCGATGCCCTCGGTGGTTTTCGCCGGAGCTCGCTCGGCTTCTTACTCGGCTCCTCGATACGAACCC
GGAAACCAGAATTACAATTCCGGAGATTATGGAGAATCGGTGGTTTAAGAAAGGTTTTAAACATATTAAATTTTACATTGAGGATGATACGCTTAGACTCTGTAATGCTGAGGAGGCTGAGGATGATGTGGA
GTCTTTGTCGGATCAATCTATGTCAGAGTCGGAGTCGGAGTTGGAGACTCGGAGGAGGCTTTCTACATTGCCAAGACCGGCGAGTTTGAATGCATTTGATATTATATCATTTTCACCAGGTTTCGATCTCTCTG
GATTGTTTGAGGAGGGAGGTGGAGACGCGCGATTTGTGTCGGGTGCACCTGTATCGAAGATTATATCAAAATTAGAGGAGATTGCCAAGGTGGTGAGCTTTACAGTGAGGAAGAAGGATTGTAGGGTGAGCT
TAGAGGGATCCAGAGAGGGGGTGAAGGGGCCATTAACGATTGCAGCTGAGATTTTTGAGTTAACGCCCTCATTGGTGGTGGTGGAAGTTAAGAAGAAAGGAGGCGACAAAGGGGAGTATGAGGAGTTCTGT
AATCGTGAATTAAAACCCGTGTTGCAGAACCTTGTTGATGACTCTGCTGCTTCTTCACATTTTCAAAACTCCTTGCACAAAGGAGAGTGTGGGCAACTTTCTAATCTGGAATTGGAGTCTGGATTGAAGAACCT
TGTGGTGGATGACTCTGCTTCTACACCACATTTGCCCTCAGATACTGAATAG
>**VvCIPK21-1**.V.vinifera v2.1|VIT_211s0052g01700.2
ATGGGAGTGGCTAATAACATAGGGAAGTACCAGCTGAGTAGGACGATCGGAGAAGGTACTTTTGCCAAGGTTAAGTTGGCAGTGAACACCGAGAATGGCCAGTATGTTGCCATCAAGATCATCGACAAGCA
CATGGTCATGGAGAACAATCTCATCTTTCAGGTACAGAGAGAGATAAGAACGATGAAGCTTCTGCATCATCCCAACATTGTTAGGATACATGAGGTCATTGGCACCAAGACAAAGATTTACATAGTAATGGA
ATATGTATCAGGAGGGCAGCTCTCAGACAAGATGTCTTACGTCAAGAAGCTAGATGAACAGGATGCTAGAAAACATTTCCAGCAGTTGATTGATGCAGTGGACTACTGCCATGGCAGAGGGGTTTCTCATAG
AGATCTAAAGCCAGAGAACTTGCTTTTGGACAGCAAAGGAAATGTAAAAGTCTCGGATTTTGGACTCAGTGCATTGCGCAAGCCTGGAGATGTGTTATCCACAGCATGCGGTTCACCATGCTATGTAGCACC

AGAGCTGCTTGCAAATAGGGGCTACGATGGAGCAGCTGCAGATATTTGGTCTTGTGGAGTAATCCTTTTTGAGTTACTTGCTGGTTATCTTCCATTTGATGATCGCAATTTGATGAGCTTGTATAAGAAGATAA
CAAAAGCAGAGTACACTTGTCCAGAGTGGTTCACACCAAGCCAAACAAAGCTAATCTCCAGGATCCTTGAGCCCAACCCTAGACGGCGAATATCAATAGCAGAGATTGTTGAAGATGAGTGGTTTCAGATTG
ACTACGAACCTGCTTCTGGAATTGAATTTGATGACAAAATCAACCTAGATGATGTTCATGCTGCTTTCAATTTGATTGAGGAAAATAACGCCGAAGCAAAGGTACCCCACTCAAGTTTCATTAATGCTTTCCA
ACTAATAGCAATGTCACATGACCTCGATTTGTCAGGTCTTTTTGAAGAGAAGGATGACAACAAGCAGAAAGCAAGGTTTGGATCCAAGCACACAATTGATGAAACTATAAAGAAAATTGAGGCCGCTGCAA
AGGATGTGAGCCTCTCAGTTGAAAGGAAGAACTCTAAGATGAAGATGCATCCACAACAGAAGATGACTAGATGTTCTCGATCATATTTCAACCTATCAGCAGAGGTGATTGAGGTGGCTCCCACTCATTGTG
TTGTAGAAATATCAAAATCTGCAGGCGAGCTTGGTGTGTACAAAGAGTTCTGCACAAGCCTATCATGTCTGCTAAAAGAGAAATCGGGCATTCCATTAGAAGAAAAAGATTCCAATGAAGATGTCGTTAACA
TCAAAGATATTCAGGAAACTGAATGCTCTGAAAAGAAAGATGACAAGGACGACAAAGACATCAATGGCTATTCTTCCTCTTGA
>**VvCIPK21-2.**V.vinifera v2.1|VIT_211s0052g01700.3
ATGGGAGTGGCTAATAACATAGGGAAGTACCAGCTGAGTAGGACGATCGGAGAAGGTACTTTTGCCAAGGTTAAGTTGGCAGTGAACACCGAGAATGGCCAGTATGTTGCCATCAAGATCATCGACAAGCA
CATGGTCATGGAGAACAATCTCATCTTTCAGGTACAGAGAGAGATAAGAACGATGAAGCTTCTGCATCATCCCAACATTGTTAGGATACATGAGGTCATTGGCACCAAGACAAAGATTTACATAGTAATGGA
ATATGTATCAGGAGGGCAGCTCTCAGACAAGATGTCTTACGTCAAGAAGCTAGATGAACAGGATGCTAGAAAACATTTCCAGCAGTTGATTGATGCAGTGGACTACTGCCATGGCAGAGGGGTTTCTCATAG
AGATCTAAAGCCAGAGAACTTGCTTTTGGACAGCAAAGGAAATGTAAAAGTCTCGGATTTTGGACTCAGTGCATTGCGCAAGCCTGGAGATGTGTTATCCACAGCATGCGGTTCACCATGCTATGTAGCACC
AGAGCTGCTTGCAAATAGGGGCTACGATGGAGCAGCTGCAGATATTTGGTCTTGTGGAGTAATCCTTTTTGAGTTACTTGCTGGTTATCTTCCATTTGATGATCGCAATTTGATGAGCTTGTATAAGAAGATAA
CAAAAGCAGAGTACACTTGTCCAGAGTGGTTCACACCAAGCCAAACAAAGCTAATCTCCAGGATCCTTGAGCCCAACCCTAGACGGCGAATATCAATAGCAGAGATTGTTGAAGATGAGTGGTTTCAGATTG
ACTACGAACCTGCTTCTGGAATTGAATTTGATGACAAAATCAACCTAGATGATGTTCATGCTGCTTTCAATTTGATTGAGGAAAATAACGCCGAAGCAAAGGTACCCCACTCAAGTTTCATTAATGCTTTCCA
ACTAATAGCAATGTCACATGACCTCGATTTGTCAGGTCTTTTTGAAGAGAAGGATGACAACAAGCAGAAAGCAAGGTTTGGATCCAAGCACACAATTGATGAAACTATAAAGAAAATTGAGGCCGCTGCAA
AGGATGTGAGCCTCTCAGTTGAAAGGAAGAACTCTAAGATGAAGATGCATCCACAACAGAAGATGACTAGATGTTCTCGATCATATTTCAACCTATCAGCAGAGGTGATTGAGGTGGCTCCCACTCATTGTG
TTGTAGAAATATCAAAATCTGCAGGCGAGCTTGGTGTGTACAAAGAGTTCTGCACAAGCCTATCATGTCTGCTAAAAGAGAAATCGGGCATTCCATTAGAAGAAAAAGATTCCAATGAAGATGTCGTTAACA
TCAAAGATATTCAGGAAACTGAATGGTAA
>**VvCIPK24**.V.vinifera v2.1|VIT_211s0016g05420.1
ATGAAAAAGGTCACGAGAAAGGTCGGAAAGTATGAGGTCGGCCGAACAATTGGCGAAGGAACTTTCGCCAAGGTTAAATTTGCGCACAACACCGAGACTGGGGAGAGCGTAGCTATGAAGGTTATGGCAA
AGTCCACCATTCTTAAGCATAGGATGGTCGATCAGATTAAACAAGAGATATCCATAATGAAGATTGTCAGGCACCCTAACATAGTTAGATTGCATGAGGTCTTGGCCAGCCAGACAACGATATATATTATTCT
TGAGTTTGTCACTGGAGGAGAACTATATGATAAAATTGTTCACCAAGGAAGACTCTCTGAAAATGAATCCAGGCGATACTTTCAGCAGCTTATAGATGCTGTATCACATTGTCACAGTAAGGGTGTGTACCAC
AGAGATCTGAAGCCTGAAAATCTACTTCTTGATTCGAATGGAAATTTGAAGGTTTCTGATTTTGGACTTAGTGCATTGCCTAAACAAGGGGTTGGACTTCTTCATACCACATGTGGAACCCCAAATTATGTTGC
ACCTGAGGTGCTTAGTAACCAAGGTTATGACGGTGCAGCTGCTGATGTGTGGTCATGTGGAGTCATTCTCTATGTTCTAATGGCAGGATATCTTCCATTTAATGAGATAGACCTTCCAAGCTTGTACAAAAAG
CGTATTCGGATTGAAGGGATAAAAAATGATCCATGGTTTCGAAAGAACTACGTGTCTAGCAAATGTAGAGAAGATGAAGAAGTGAATTTGGATGATATTCATGCGGTTTTTGATGATATTGAGGAAAATTAC
GTATCTGAGCAGTCAGGAAATAATGACACAGGTCCTTTGATAATGAATGCATTTGAGATGATTACCCTATCCCAAGGGTTGAATCTATCAGCACTTTTTGACAGACGACAGGATTATGTAAAACGGCAAACTC
GTTTTGTTTCCCGCGAACCAGCAAAAGTTATAATTTCTACTATTGAAGCTGTGGCAGAATCTATGAATTTCAAGGTCCATACACGCCACTACAAGACAAGACTCGAGGGAATATCTGCAAATAAGACTGCACA
ATTTGCAGTTGTCCTAGAGGTTTTTGAAGTTGCACCATCCCTTTTCATGGTGGATGTTAGAAAGGCTGCTGGGGATACACTTGAATATCACAAGTTTTACAAGAATTTCTGTGCTAAACTTGAGCATATCATCT
GGAAACCAAAAGAGGCTATGACAAATTCCAGTCTTCTAAGAACAATGACTTGCTGA
>**VvCIPK27**.V.vinifera v2.1|VIT_208s0058g01090.1
ATGAGCAGTGAGCACCACCGGTCCACCACATCAAGGCGCTTTGTTCCTTCTCCGGAAATTGTGGTTTCAGAGATGGAGAACGATACTGGAGATGCTGATCCGGCGAACGAAGATATTCAGCCGGAGATAACT
TTGTTCGGAAAGTACGCGGTTGGAAAGTTGCTCGGCTATGGAGCCTTCGCGAAGGTCTACCACGCAAGGAACATCCGAACGGGCCAAAGCGTGGCGATCAAGGCGGTGAGCAAGAAAAAGGTGATGAAAGG
AGGATTGACGGCCCATGTGAAGCGGGAGATTTCTATCATGCGCCGGCTACGGCATCCTCACATCGTTAAGCTGTATGAGGTCTTGGCGACGAAAACGAAGATCTACTTCGTGATGGAGTACGCCAAAGGTGG
TGAGCTCTTTACAAAAATAGCCAAGGGGCGGTTCAGCGAAGACCTCAGTCGCCGGTATTTTCAGCAGCTGATCTCCGCCATTGGATATTGCCACTCCCGAGGCGTTTTCCACCGCGATTTGAAGCCGGAGAAC
CTTCTTCTCGACGAGAATTGGAACCTTAAAGTCTCCGATTTCGGTCTCAGCGCCGTGAAGGATCAGATCCGGGCCGATGGTCTCCTACACACTCTCTGCGGTACCCCGGCGTATGTGGCGCCGGAGATTCTAG
CGAAGAAAGGTTACGATGGTACCAAGGTGGATATCTGGACTTGCGGCATCATTCTCTATGTATTGAACGCCGGTTATCTTCCGTTCAACGATCCAAACCTCATGGGAATGTACCGGAAGATCTATAAAGGCGA
GTTTCGGTGTCCGAAATGGACTTCTCCTGATATGCGACGCCTTCTATCACGCCTTCTCGATACGAATCCAGAGACGAGAATCACTGTCGATGAGATCATCCGTGACCCTTGGTTCCAAAAGGGTTACAAGGAA
ATTAAATTCCACGTGGAAGATTTTGATTTCAAGGATCACGACGACGACGACGACAACGCCGAGTACATGAACGCGTTCCACATTATCTCATTCTCGTCCGGTTTCGAATTCTCCAGTATGTTGAACGATTCCG
GCATTTCGGTGGCCGGTGAAAGATTCGTATCGGCCGAGTCACCGGAGAAAATAATAGAAAAGGTCGAGGACGTGGCAAAAAAGGAGAACGTAACAGTGACGAGAAAGGAAAATTGGGGAGTGATGTTGGA
GGGACAAAACGGTAATTTCGCCTTGATAATGGAGATATATCGGTTGACGGAGAAGCTGGTCCTGGTAGAGGTGAGGAAGAAAGAGGTGGACGCTGGACTAGGACCTGGCAAAGAAATCTGGCGAGACACAC
TCCGGCCGCAGCTCAGCGAACTGATTTATCAACCGGAGGAGCCGAGCACCGAGAGCTGA
>**VvCIPK28**.V.vinifera v2.1|VIT_206s0004g07870.1
ATGCCAGAGATCGAACGCGGCTCAGGGAGTGGGGCCGCTTCGGCTACCTCGTCGGAGAAGGCTTTGTTTGGGAAGTATGAGCTCGGCAGATTGCTGGGCTGTGGGGCGTTTGCGAAGGTGTATCATGCGAGG
AATATCAGGACAGGGCAAAGTGTGGCCATTAAAGCAATATGTAAGAAGAAGATCGTTGGGAGCAACTTGATGGCGAATGTGAAACGGGAGATCTCTATCATGCGCCGGTTGCAGCACCCTCACATCGTGAA
GCTGTATGAGGTGTTGGCGACCAAGACCAAGATCTATTGCGTGATGGAGTTCGTGAAAGGTGGGGAGCTGTTCGCGAAGGTCGCCAAGGGGCGGTTCAGCGAGGATCTGAGCCGTCGGTATTTCCAGCAGTT
GATCTCCGCCGTCGGGTACTGCCATTCGCGGGGGGTGTTTCACCGGGATCTGAAACCGGAGAACCTCCTCGTTGACGAGAACGGGTGCTTGAAGGTTTCAGATTTCGGGCTCAGCGCCTTGACGGATCAGATT
CGCCTCGACGGCCTCCTGCACACCTTGTGCGGCACTCCAGCTTACGTCGCGCCGGAGATTCTCACCAAGAAAGGCTACGATGGGGCGAAAGTGGATGTGTGGTCCTGCGGAGTCATCCTCTTCGTGTTGAACG
CCGGTTACCTACCCTTCAACGATCCCAATCTCATGGCCATGTACAAGAAAATATACAAAGGCGAGTTCCGGTGCCCTAAATGGATGTCGCCGGACCTTAAACGTTTCCTTTCTCGCCTCCTCGACACCAACCCC
AACACCAGGATCACCGTCGATGAAATCCTGCAGGACCCATGGTTCAGAAAAGGATACAAAGAGGTGAAGTTCCATGAAGAAGATTTCGGACTCAAATCCGAAAAAGAGGACCGAAGCCCCACGAACTTGAA
TGCCTTCGACATAATTTCGTTCTCGTCTGGTTTGAACTTGTCGGGTCTGTTCAATGACTCGTCCAAGTCGTCGCCGGATGACGACGAGCGATTTGTATCGGCCGAGCCCGTGGAGAAAATAATCGAGAAAATC

GAGGAAATTGCCGAAGGTGAGAACCTGACGGTGAATTGGAAGAAGGAATGGGGAGTTGACCTGCAGGGACCAAATGGTAATTTCATAATCGGGGTTCAAATTGATCGATTAACCCATGATTTGGTCGTCGTG
GAGGTTAGGAGGAGAGCCGGCGATGCAGCGGCCTACGCCGCCGCGTGGAAGGATAAGCTCCGGCCGGCGCTCGCCGGACTCATGTTCCAAGGACCGGAGACCCAAGTCGCCGGCGATTAA
>**VvCIPK29**.V.vinifera v2.1|VIT_205s0020g04570.1
ATGGAACCTACCCCTGCCGGGGTTATCCTTGGCAAGTACAAACTCGGCCGCCTTCTCGGCCGAGGCAGCTTCGCCAAGGTGTACCAGGCCCACTCCATCACCGACGGCACTGCCGTGGCCGTGAAGATCATG
GACAAGGCACGAAGTGTTGAGGCCGGCATGGAGTCTCGGATCGTTCGCGAAGTGACTGCCATGCGGAGCCTTCAGCATCCCAACATCCTCAAAATCTACGAGATCATGGCCACTAAGTCCAAGATTTACTTG
ATTATTGAGTTGGCCAAAGGAGGCGAACTCTTCACCAAAATCTCCCGCCGCGGGCGCCTCACTGAGGCAGCGGCGCGCCGTTACTTCCAACAACTGGTATCGGCTCTCTTGTTCTGCCACCAGAACGGCGTGG
CTCACCGCGACATGAAGCCGCAGAACCTTCTCCTGGACCAGGACGGTAACCTCAAGGTCTCCGACTTCGGGCTGTCCGCGCTTCCGGATCAACTTAAGAACGGCCTGCTCCACACAGCTTGTGGCACGCCTGC
GTACACGGCGCCGGAGGTCGTTTCTCGGAAAGGCTACGACGGCGCAAAGGCGGACGCGTGGTCTTGCGGAGTCATACTTTTCGTCTTTCTTTCTGGCTTTCTCCCGTTCGATGATAGTAATCTCGTGACAATGT
ACCGGAAAATCCATCGTCGGGATTACCAGTTTCCGTCTTGGATCTCCAGGCCGGCGCGATCGGTAATATCACAGTTGCTCGATCCGAATCCTAATAAAGGATGAGCATCGAAGCGCTGATGGAATCTGCCTG
GTTCAAGAAATCTACGCCGGCGAAAAAACTTGAACATGCGAGTTCGTTTGATTCAGACATGGAATCAATGAAAGTTTGTGAATATGGTTCAGCGCCGCTAACCATGAATGCATTCGACATAATATCGATGTCG
TCTGGGTTGGACCTGTCGGGGCTTTTCTTCGAAGCAGCAGCGAATAAGAGGGAGAAGAGGTTCATGTCGAGGGCATCAGCAGAGATAACTGTGGAGAAGATGAGAGAGGTTGGTGAGAAATTGGGATACGG
TGTTGAGAGAGGGAAGGTTGGAAGTATAAGGAGGGAGGCGGGAAGGGTGGTTCTGTTGGTCGAAGTTTCAGAAGTTGCTCCATCATTGTTACTAGTGGAGGTGCAGGTGGTAGACGGAGGAGAATTTGAGG
AGTTGAAATGGGAAATTGTGAAAGCTGGGCTTGAAGATATAGTTGTTTCATGGGAGGGTAGTGGGATGTAG
>**VvCIPK30**.V.vinifera v2.1|VIT_209s0070g00160.1
ATGGCTAAGATTAGCCAAGACAAGTTAGACTACTCAAGCCTGCTCCATGGCAAATACGAGCTAGGCCGCCTGCTTGGCCATGGCACCTTTGCAAAGGTGTACCATGCCAGGAACTTAAGAAATGGGGAGAGT
GTAGCCATGAAAGTGGTTGCGAAAGACAAGGTGATAAAGGTGGGGATGATGGAGCAAATGAAGCGTGAGATATCGGTGATGAAGAAGGTGAGCCACCCCAACATCGTCCAGCTCCATGAAGTCATGGCAA
GCAAGTCCAAGATATACCTGGCCATGGAATATGTTCGTGGGGGTGAGCTCTTTTCCAAGATTGCCAAAGGCAGACTGCGGGAGGATGCAGCCAGGTCCTATTTCCAACAATTGATTTCAGCCGTGGAGTTCTG
CCACAGCCGCGGGGTTTTTCACCGGGACTTGAAGCCTGAGAACCTCCTCCTGGATGAACAGGGGAACCTCAAGGTCACTGATTTTGGGCTGTGCGCGCTGTCGGATCACTTGAAGCAGGATGGTTTGCTGCAC
ACCGCCTGTGGGACTCCGGCCTATGTTGCGCCGGAGGTGATTGCGAAGAGGGGCTACGATGGCGCAAAGGCAGATATATGGTCTTGTGGGGTGATACTGTTCGTGCTTATGGCTGGATTTTTGCCCTTCCAGG
ATGACAACCTTATTGCTATGTACAAAAAGATCTACAGAGGGGACTTCAAGTGTCCGCCTTGGTTCTCACCTGAAGCGCGCAGAGTTGTTTGTAGGCTGCTTGATCCAAACCCCAATACCAGAATCACCATTTC
CAAGCTCAAGGAAACATCCTGGTTTAAGAAAACAAAGCCTAAAAATGTGCAGACTACTGCTAGTGGTGTTGCCGTCGAAGAGGAAATGGAAGTGAAAGAGAAGGTGCCTGAGGCTCTTAACGCCTTTCACA
TAATCTCGTTATCGGAGGGGTTCGATCTGTCGCCTCTGTTTGAGGAGAAGAAGAAGGAGGAGGAAGAGGAGATGAGGTTTGCGACGTTAAAAAAGTCGATGAGCGACGTGGTATTGAAGCTAGAGGAGCTG
ACCAAGCCTGGGATGTTTCCGATGACTCCAGCTCTCGGAGGAGACAGTGTGGATCAGTACAATCAGAGTTGCGGCAAGGAGTTGAGACCAGCAGTGGAGGGCATAGGCTGGACAGCAACCCAAAATTCGGC
AGTTGCTTGA
>**VvCIPK31**.V.vinifera v2.1|VIT_211s0016g00200.1
ATGGCGGATTCGGGTAAAGATTCGTGCCCGTCTTTGCTTCACGGTAAATACGAGCTGGGACGGCTTCTGGGTCACGGCACCTTCGCCAAGGTGTATCATGCCCGGAACTTGCAGACAGGGAAGAGTGTAGCG
ATGAAAGTGGTGGGTAAGGATAAGGTGATAAAAGTGGGGATGACGGAGCAGGTGAAGAGGGAGATATCAGTTATGAAGATGGTACAGCATCCCAATATTGTTGAGCTTCACGAAGTCATGGCCAGCAAGTC
CAAGATCTACTTCGCCATGGAGCTCGTCCGCGGCGGCGAGCTTTTCTCCAAGATCTCCAAAGGGCGGTTGAGGGAGGACGTTGCTAGAGTGTACTTCCAGCAGCTGATCTCGGCGGTGGACTTTTGCCACAGC
CGCGGAGTCTACCACCGGGATCTGAAGCCGGAGAATCTACTCCTCGACGAGTCTGGCAACTTGAAAGTCACCGATTTTGGGCTGAGCGCGCTGTCAGAGCACTTGAAGCAAGATGGGCTTTTGCACACGACA
TGTGGGACTCCAGCCTACGTCGCGCCCGAGGTGATTGGAAAGAATGGGTATGATGGGGCAAAGGCCGATCTGTGGTCGTGCGGTGTGATTCTGTACGTTCTTCTCGCCGGATTCTTGCCGTTTCAGGACGACA
ACATTGTGGCCATGTATAGGAAGATCTACAGAGGAGACTTTAAGTGTCCGCCTTGGTTTTCACCCGAATCTCGCAGACTCGTCACGAAACTCCTCGATCCAACCCCCAACACTCGAATCACCATAGCCAAGAT
CGTGGAATCCTCCTGGTTCAAAAAGTCGGTGCCCAAAACGATACTAACCAAAGAGGAACAAGAATTCGAGTCATTCAATTGCGGAAAAGCAAAGCAGCCGGAGACCCTGAACGCGTTCCACATCATCTCCCT
ATCGGAGGGGTTCGATCTGTCACCTTTGTTCGAAGAGAAGAAGAGAGAGCAAAAGGAGGAACTGAGGTTCGCGACGACGCAGCCAGCGAGCAGCGTGATATCGAAACTGGAGGAGGTAGCGAAGGCAGGG
AAATTCAGCGTGAAGAAGAGCGAAACGATAGTGAGGCTGCAGGGGCAGGAAAGAGGAAGAAAAGGGAAATTAGCGATAGGAGCGGAGATATTCGCAGTGGCGCCGTCTTTCCTGGTGGTGGAGGTGAAGA
AGCACCATGGTGATAGCCTGGAGTACGACCAGTTCTGCAACAAGGAGCTCCGGCCGGCTCTCAAGGACATAGTTTGGACGTCCACCCCAGCCTGA
>**VvCIPK32**.V.vinifera v2.1|VIT_204s0008g05770.1
ATGGAAGAGGTCAGGAGTATGCTGTTTGGGAAGTATGAAACGGGGAGGTTGCTCGGGAAAGGGACCTTCGCCAAGGTCTACTATGCTAAGCATATGGTGTCCGGGGAGAGCGTTGCGATCAAAGTAATCAA
CAAAGACTATGTGAAAAAGGAAGGAATGATGGAGCAAATCAAACGAGAAATATCGGTGATGCGATTAGTACGACACCCCAATATCGTTGAGCTCAAAGAAGTGATGGCCACAAAGACCAAGATCTTCTTTG
TGATGGAGTACGTTCGCGGCGGCGAGCTATTCGCCAAGGTCGCCAAGGGGAAACTGAAAGAAGATCAGTCCAGAAAGTACTTTCAGCAGCTCATCAGCGCTATCGATTTCTGTCACAGCCGTGGCGTCTCTC
ACCGTGATCTCAAGCCGGAAAATCTTCTCCTGGATGAAAACGAAGACCTTAAAGTGTCTGATTTTGGGTTGTCGGCGTTGCCGGAGCAGCTCCGGAACGATGGACTTCTTCACACACAGTGTGGGACTCCGGC
ATATGTCGCGCCGGAGGTTTTGAGGAAGAAAGGGTATGATGGGGCCAAAGCAGATATGTGGTCTTGTGGGGTTATCCTCTTTGTACTTCTTGCAGGGTTTCTACCTTTTCAAGAAGAGAATCTGATGAAGATG
TATAGGAAGGTTTTCAAGGCTGAATATCAGTTCCCACCATGGTTTTCATCAGAAGCCAAAAGACTAATATCAAAGCTCCTGGTAGCTGACCCTCAAAGACGGATAACAATACCGGCAATAATGCGAGTTCCCT
GGTTCCGAAAAGGGTTTTCAAGGCCTGTTGCTTTTTCAATAGAAGAACCATTCCCGGACAAGCCTGAGGACAGTGATACACTAAAGCTTGAAGCTGCAAAGAGTACCAATTCCCCGTCCCCGGCCTTCTTCAA
TGCATTTGAGTTCATTTCCTCGATGTCATCGGGCTTCGACTTATCAAGCTTGTTTGAAAATCAGAAGAAGGCTGGATCAATGTTTACCTCCAAGTGCTCGGCGGCTGCGATCATGGCCAAAATTGAGCTGGTGG
CCAAGGGTTTGAAGTTTAGGGTTGCAAAGGTGAAGGACTTCAAGTTGAAGCTTCAAGGGCAATCAGAGGGCCGGAAAGGGAGGTTATCGTTGACGGCGGAGGTGTTCGAGGTGGCGCCTGAAGTCGCCATC
GTCGAGCTCTTGAAGTCGGCTGGCGATACGTTGGAGTATGCCAAATTCTGTGAGGAAGACGTTAGGCCTGCATTGAAAGACATTGTTTGGACATGGCAAGGTGACAATCACAATGGTAATGTTAGCAACAAT
ATTGTTGAATGTGAAAATTAA
>**VvCIPK33**.V.vinifera v2.1|VIT_209s0070g00140.1
ATGGAGGGGCAGAAGAACCAAGTTGTTGATGAAGCAAGAAGCATATTGTTTAAGAAATACGAGATGGGAAGGCTTCTTGGGCAGGGCACCTTCGCAAAGGTGTACCACGCGAAGAGGCTTGGGAGTGGGGA
CAGCGTTGCCATCAAGGTGATCAACAAGGACCACGTGAAGAAACAGGGCATGATGGAGCAGATCCAGCGCGAAATCTCTGTTATGCGCCTTGTTCGCCACCCCAATGTGGTGGAATTGAAGGAAGTGATGGC
CACAAAGACCAAAATCTTCTTCGCCATGGAGTTCGTAAAGGGTGGTGAATTGTTCGCAAAAGTGACCAAGGGAAGGCTCAAAGAGGATTCGGCTAGAAAATACTTCCAGCAGCTTGTCAGCGCCATCGATTT

TTGCCACAGCAGAGGCGTTTCCCACCGCGATCTCAAACCCGAAAACTTGCTCCTCGATGAAAACGAGAACTTGAAAGTCTCCGATTTTGGGCTGTCGTCGTTGCCGGAGCAGCTCAGAAATGATGGATTGCTG
CACACACAGTGTGGGACGCCAGCATACGTGGCGCCGGAGGTGTTGAGGAAGCGAGGCTACGACGGAGCCAAGGCCGATACATGGTCTTGTGGGGTTATTCTCTATGTTCTGCTTTGCGGCTTTCTTCCATTTC
GAGCAGAGAATGTTATGAAGATGTACACCAAGGTTTTCAAGGCAGAGTTCGAGTTTCCTCCATGGGTTTCGACCCAAGCAAAGCGGCTGATATCGAAGCTCCTCGTTGCTGATCCCGAGAAACGAATCACCA
TTTCTGCGATCATGGAGGATCCTTGGTTTCAGAAAGGTTTCAGGAAGCCTATTGCCTTTTCAATTGAAGAACAAGAGAACAACGAGGAGTGGCAAGGGTGTAGAGGGGAGGTGGAAATGGTGAGAACAAAG
TCCTCACCACCTTTCTACAACGCCTTCGAGTTCATCTCCTCAATGTCATCGGGCTTCGACTTGTCCAGCTTGTTCCTGAGCAAGAGGAAGTCGGGCTCCATGTTCACCTCCAAGTGCTCCGCCTCAACCATCATG
GCCAAGCTCGAATCACTGGCTAAGACGCTCAATTTCAGAGTGGTGAACGTGAAGGAGTTCAAGGTGAAAATGCAGGGGCTAGTGGATGGGCGGAAGGGGAAGTTGTCGGTAACGGCCGAGGTGTTCGAGGT
GGCGCCGGCAGTGGCCTTGGTCCAATTCTCCAAGTCCGCCGGTGACACCCTGGAGTACACCAAGTTTTGTGAGGAGGAGGCCAGGCCTGCTCTCAAAGACATTGTTTGGAGTTGGCAAGGTGAAAATGAGTG
CATAAATGATAATTTGTTCTCTGTTTGTATTTTTGGCGAGTCTATGACATGTGAGGAATGGTAG
>**VvCIPK34**.V.vinifera v2.1|VIT_208s0058g01040.1
ATGGAAAATAATGGGAGTATCCTGATGCAACGGTATGAATTAGGGAAATTATTAGGTCAAGGCACCTTTGCCAAGGTCCATCATGCCAGGAACCTTAAAACTGGCACCAGTGTTGCCATTAAGATAATTGAT
AAAGAGAAGATTTTGAAGGTTGGGATGGTGGATCAGATTAAGCGAGAGATTTCTGTAATGAGACTGGTTAGACACCCAAATGTGGTAGAGCTTTATGAGGTTATGGCCAGCAAAACGAAGATTTACTTTGTC
ATGGAATATGCTAAAGGAGGGGAGCTCTTCAACAAGGTAGCCAAAGGAAAGCTCAAGGAGGATGTTGCAAGGAAATATTTTCAACAGCTGATCAGTGCGGTTGATTTCTGTCACAGTAGAGGTGTTTATCAT
CGGGATTTAAAGCCAGAAAACCTTCTACTGGATGAGAATGGAAATCTAAAAGTTTCAGACTTTGGACTGAGTGCCCTTGCTGAATCTAAGCACCAAGATGGGTTGCTTCACACAACCTGTGGAACCCCCGCCT
ATGTTGCTCCAGAGGTGATCAATAGGAAAGGCTATGATGGAGCCAAAGCTGATATATGGTCATGTGGGGTGGTCTTATTCGTTTTGTTGGCTGGCTATCTCCCATTTCATGATTCAAATCTGATGGAGTTGTAT
AGGAAGATTGGTAAGGCAGAATATAGATTCCCTAACTGGTTCCCTTCGGAAGTACGAAGGTTGCTGTCAAAGATCTTGGATCCAAACCCAAAGACTAGGATTTCCATTACCAAAATAATGGAAAATTCTTGG
TTCCAAAAGGGTCTTGATCCTGTCCTGAGGATCACTGAAACAGGAGAGAAAGAGCCAGCTGATCTCGAGGTTGAGGCTGTATTTGGTTCTAATGAGAGTGCCAGCACCACTGCTGAGGCAAAGCAAGAATTG
GCGACGCTTAGTAACTTGAATGCGTTTGATATCATTTCCTTTTCAGCAGGGTTTGACTTGTCTGGTTTGTTCGAGGAAAATAAGCAAAAGAAAGAAGTGCGATTTACATCTAGGCAGCCAGCCTCAGCCATCA
TCTCTAAACTGGAGGACATTGCTAAGCGTTTGATGCTGAAGGTGAAGAAGAAGGATGGTGGGTTGTTGAAAATGGAAGGTTCAAAGGAAGGCAGGAAAGGCGTCTTGTCCATTGATGCAGAGATCTTTGAG
GTGACTCCATCTTTTCATTTGGTGGAGGTGAAGAAGTCCAGTGGGGACACGTTGGAGTACCAGAAAATAGTGAAACAGGAGATAAGACCAGCTCTCAAGGACATTGTGTGGGCATGGCAAGGTGAGCAGCA
GCAGCAGCAACAACAACCAGAAGAGCCAATGCCCCTATTGCAGCCCATCTCACCTCCAGCTTCATAA
>**VvCIPK35**.V.vinifera v2.1|VIT_216s0022g00350.1
ATGGATGATCTGCCTCTATGGTTAGATGTGAATGAAGATCCAAGTAATGATTATTTTGTGGAAAACCTTTGGCATCATCCTAATCAACTGTCAGAAGAAATGGTGCACATGAGAGACATATTCCTCTTTTTAAC
AGATTCGTCAAAGCTTTTTTCCTCTGAGGGTGTGGCTTCACCTCGAGAGCATTCCTGGAAAGGTAGATTGGACAAGAAGTACAGGAACCTTAAAACTGGCACTAGTGTTGCCATTAAGATAATTGATAAAGAG
AAGAATTTGAAGGTTGGGATGGTGGATCAGATTAAGCGAGAGATTTCTGTAATGAGACTGGTTAGACACCCAAATGTGGTAAAGCTTTATGAGGTTATGGCCAGCAAAATGAAGATTTACTTTGTCATGGAA
TATGCTAAAGGAAGGGAGCTCTTCAACAAGGTAGCCAAAGGAAAGCTCAAGGAGGATGTTGTAAGGAAATATTTTCAACAGCTGATCAGTGCAGTTGATTTCTATCACAATAGAGGTGTTTATCATCGGGAT
TGA
>**VvCIPK36**.V.vinifera v2.1|VIT_210s0003g01410.1
ATGGAGGAATTGAGTCCATGTTCAGAAGCATGTGGACCAGCAATCCCCATGGAGGCCACCCACCACTACGTGCCTCTCTCTCCGTACGAGATGGGGAGAATGCTTGGGCAAGGGACTTTTGCCAAGGTTTAC
CATGCTCGGAACCTCAAGTCCGGTCAGAGTGTTGCCATAAAGATGATTGACAAAGAGAAGGTGTTGAAGGTGGGGTTGATAGATCAAATCAAGAGGGAGATCTCGGTGATGCGCCTTGTTCGACACCCTAAT
GTTGTTCAACTCTATGAGGTCATGGCGAGCAAGACCAAGATTTATTTTGTCATGGAGTATGTCAAAGGTGGAGAGCTGTTCAACAAGGTGGCGAAAGGCAAACTCAAGGAAGACGCGGCTCGCAAGTATTTC
CAGCAATTGATTGGAGCTGTTGACTTCTGCCATAGCCGAGGAGTCTACCACCGCGACCTCAAGCCGGAGAATCTCCTCCTTGACGAGAATGGAGACCTTAAAGTAACCGATTTTGGCTTGAGTGCGCTGTGGG
ACTCAAGGAGGCATGATGGCCTCCTCCACACTACTTGTGGAACTCCAGCTTATGTGGCTCCGGAGGTCATCAACAAGAAGGGCTATGATGGAGCCAAGGCAGACATGTGGTCATGTGGCGTCATTCTCTTCGT
TCTCATGGCTGGTTTTCTCCCTTTCCATGACCCTAATCTCATGGAAATGTACAGGAAGATCAGCAGAGGTGAATTCAAGTGCCCACAATGGTTCCCACTGGAGGTCCGGAAGCTTCTTTCTAAAATTCTCGACC
CCAGCCCCAGCACTAGAATAACTGTGGCCAAGCTCATGGACAACCCTTGGTTCAAGAAGGGATTCAAACCGATTGTAGCCCCACAACCCAATCGAAATGATTCGATGAGCGATGTCCGTGCTGTATTTGCCA
CACATCCAGAAGGAACTTCTGAGGAGGAAGCCGAGGAAACAACAAGCAGCATGAAGCCAACTTGTTTGAATGCCTTCGATATCATCTCTCTCTCACCCGGGTTTGATCTGTCAGGTCTGTTTGAGAAAGATAC
CAATCATAGGCCAGACGCTCGGTTCACCACCAAACAGCCTGCATCAACCATTGTATCCAAACTGGAAGAAATAGCAGAAATGGAGAGATTTAAGGTTAAGAAAAAGGATGGGACGGTGAAGTTGCAAGGCA
GCAAGGAAGGGAGAAAGGGTCAGCTTGCTATAGATGCTGAAATTTTCGAGGTCACGCCTTCACTTTTCGTGGTGGAGGTGAAGAAAACGGCAGGTGACACCTTGGAGTATAAGAAGTTCTGTAACCAAGATT
TGAAGCCTTCACTCAAGGATATCGTATGGACTTGGCAAGGAAGTGAGCAGCAGCAGGAGCAACAGCCACAACCACAATAG
>**VvCIPK37**.V.vinifera v2.1|VIT_213s0067g02480.1
ATGGAAAATAAGGGGAATGTGCTGATGCAAAGGTATGATGTCGGGAGATTGCTAGGCCAAGGAAACTTTGCTAAGGTTTACTATGGAAGGGATCTAAAAACTGGCCAGAGTGTAGCCATTAAGGTGATCGA
CAAAGAAAAGATTTTAAAGTGGGGCTAATTGATCAGACCAAGAGGGAGATATCTGTTATGAAGCTTGTAAAACACCCTAATGTCATTCAGTTTTACGAGGTCATGGCCACCAAAACCAAGATTTACTTTGTT
ATGGAATATGCCAAAGGTGGTGAGCTGTTTAACAAGGTAGCCAAAGGAAGGCTCATGGAGGATTTTGCAAGGAATTATTTTCAACAGTTGATCAGTGCCGTTGACTTTTGCCACAGCAGAGGTGTTTATCACC
GGGATTTGAAACCAGAAAACCTATTGCTGGACGAGAATGGAGTTCTGAAGGTGACAGATTTTGGGTTGAGTGCCCTTGCAGAATCGAAGCATCAAGATGGATTACTCCACACAACTTGTGGAACCCCTGCTT
ATGTAGCGCCAGAAGTGATAAATAGAAAAGGGTATGATGGGGCCAAAGCTGACATCTGGTCTTGTGGGGTGATCTTATTTGTTCTTCTGGCTGGGTATCTCCCATTCCATGATTCAAATCTAATGGAGATGTA
TAGGAAGATAGGCAAGGCAGATTATAAATGCCCCAATTGGTTCCCATCTGAAGTGCGCAGGTTGCTGTCAAAAATCCTTGACCCAAACCCTAATACTAGGATTTCCATTGCAAAAATAATGGTAAATCCCTGG
TTTAGGAGAGGGTTCATCCCCAAGCCTTTGAAAACTAAAACAGAAGTCAAGGATTTAGATCCACTGGGCACAGATACAGTTTTTGGCCCTTGTGAGAATAACAGTGCTGCTGCCGGAGAGATGAAAGAGTTG
TCGAAACCTACCCAGCCTACCAATTTGAATGCATTTGATATCATCTCTCTTTCTACGGGGTTTGACTTGTCTGGTTTGTTTGCAACCAATGACCAAAAGAAAGAAGTACAATTTACATCCAGGCAGCCCGCCAC
AGCTATCATATCTAAACTGGAGGAGATTGCTAGGCGCCTAAGACTGAAAATTAAAAAGAAGGATGGAGGATTGTTGAAATTGGAGGGATCAGTGGAAGGTAGAAAGGGGCCATTGTCCATTGATGCTGAAA
TATTTGAGTTTACTTCGTCTTTTCATTTGGTGGAAATGAAGAAGTCCAGTGGTGATACTTTGGAATATCAGAAGATACTGAGACAGGAGATAAGGCCAGCACTCAAGGACATTGTTTGGGCTTGGCAAGGTGA
GCAGCAACAGCAGCAGCAACAACAACAACAACAAGAACCATATCAAGAACTGCAGGTTCATTCTTCTTGA
>**VvCIPK38**.V.vinifera v2.1|VIT_206s0004g07830.1

ATGGAGAGTAAAGGGGCTGTGTTGATGAACAGATATGAATTAGGAAGATTGCTAGGGCAAGGTACCTTTGCTAAGGTTTACTATGCCAGGAATCTTAAAACTGGCCAGAGTGTGGCCATTAAAGTGATAGAC
AAAGAGAAGGTTGTGAAGGTAGGGCTCAAAGATCAGATCAAGCGAGAAATATCTGTTATGAAACTGGCTAGACACCCTAATGTTTTGCAGCTTTATGAGGTTATGGCCACCAAAAGTAAGATCTACTTTGTT
ATGGAATATGCTAAAGGTGGTGAGCTCTTTAAGAAGGTGGCTAAAGGGAAGCTAAAGGAGGAAGTTGCGTGGAAATATTTTAGGCAGCTGATTGATGCTGTTGATTTCTGCCATAGCAGGGGAGTTTATCAC
CGTGATCTGAAACCGGAAAACTTATTGTTGGATGAGAATGATAACCTGAAGGTCTCTGATTTTGGATTAAGTGCCATGGCTGAATGCAAGCATCAAGATGGGCTACTCCACACCACTTGTGGGACTCCTACCT
ATGTTGCTCCTGAAGTGATCAACAAAAAAGGCTATGACGGGGCCAAAGCTGACATTTGGTCTTGTGGGGTGGTCTTATTTGTCCTACTGGCGGGTTATCTCCCATTTCATGATTCAAATTTGATTGAAATGTAT
AGGAAGATTAGTAAGGCAGAGTTTAAGTGCCCTAATTGGTTCTCACCAGATGTACGCAGGTTGCTTCAAAAGATATTGGATCCAAATCCCAATACTCGAATTTCCATTGCCAAAATCAAGGAAAGTTCTTGGT
TCAGAAGAGGATTGAAATCCAAACAGATAAAATCTGAAACAGGAAGCAAGGACATGGCTCCTTTGGATACAGATGCAGCTTCGGGTCCCTGCAAAAGTAATGGTATGGCTTCTGAAGCAAAGCAAGAGTTA
GCCAAACCTACAAACCTGAATGCTTTCGATATCATCTCTATTTCTGCTGGTTTTGATCTGTCTGGCTTGTTCGAGGAACCTTGTCAAAAGAAAAAGGCAATATTTACTTTCAGAAAACCTGCTGCTGTCATCAT
CTCTCGACTGGAAGAAATTGCCAAGCATCTGAGGCTGAAAGTGATGAAGAAGGATGCAGGTTTCATGAAATTTGAGGGCCTGAAGGAAGGTAAGAAAGGAATCTTGTCTATTGATGCAGAGATTTTTGAGGT
GACACCATCTTTTCATTTGGTGGAAGTAAAGAAGTCTAATGGAGATACGTTGGAATATCAGAAGCTACTGAAAGAAGATATAAGACCTGCTCTCCAAGATATCGTTTGGACTTGGCAAGGTGAGGAGCAGCA
GCAACAACAACAGCTTTCCTGA
>**VvCIPK39**.V.vinifera v2.1|VIT_210s0003g04020.6
ATGGCGTCACGGTCAAGTACTGGCAGGACACGTGTGGGGAAGTATGAATTGGGTCGAACCCTAGGGGAAGGGACGTTTGCAAAGGTCAAGTTTGCGAGGAATGTGGAGACTGGGGAGAATGTTGCTATTAA
GATTCTCGACAAAGAAAAGGTTCTTAAGCATAAGATGATCGGTCAGATTAAAAGGGAAATTTCAACCATGAAACTGATCAGACATCCAAATGTCATTCGAATGCATGAGGTGATGGCTAGCAAGACAAAGAT
ATACATTGTTTTGGAATTTGTGACTGGTGGTGAACTTTTTGACAAAATTGCTAGTAAAGGAAGGTTGAAAGAAGATGAAGCAAGAAAGTATTTCCAACAGCTAATTAACGCTGTGGATTACTGCCATAGTAGA
TGTGTTTTCCATAGAGACCTAAAGCCGGAGAATCTATTGCTTGATGCTAATGGAGTTCTTAAAGTTTCAGATTTTGGATTGAGTGCACTACCTCAACAAGTTCGAGAAGATGGGTTACTTCACACAACATGTGG
AACTCCAAACTATGTTGCTCCAGAGGTGATCAATAACAAAGGCTATGATGGAGCTAAGGCAGACCTATGGTCATGTGGGGTAATCTTATTTGTTCTTATGGCTGGCTATTTGCCTTTTGAAGAATCGAACCTCA
TGGCTTTGTATAAAAAGATATTCAAGGCCGACTTTACGTTTCCTCCCTGGTTATCCTCAAGTGCAAAGAAGCTAATTAAAAGAATCTTGGACCCTAACCCGGAGACGAGGATTACAATTGCTGAAGTCATTGA
GAATGAGTGGTTTAAGAAGGGATATAAGCCACCTAGTTTCGAACAAGCAGATGTTAGTCTTGATGATGTGGACGCCATCTTTGATGAATCAGGGGACTCTCAAAACCTTGTTGTGGAGAGGCGTGAAGAGCG
ACCTACGACGCCTGTCACTTTGAATGCTTTTGAACTCATCTCTACATCTCAGGGTCTCAACCTCGGTACTCTCTTTGAAAAGCCAATGGGCCTTGTTAAACGGGAGACAAGATTCACATCGAAGCGTCCTGCCA
ATGAGATTATCTCAAAAATTGAGGAAACTGCTATGCCTTTGGGGTTTGATGTAAAGAAAAATAACTTCAAGATGAAACTTCAAGGGGAGAAAACTGGACGCAAGGGTCATTTATCCATTGCAACAGAGATTT
TTGAGGTGGCCCCTTCGCTATACATGGTTGAGCTTCGAAAATCTGGGGGAGATACTTTGGAATTTCACAAGTTCTACAAGAACCTCTCAACTGGGTTGAAAGATATTGTTTGGAAATCAGGAGATGAATCAAA
GGAGGAAGTGAATAATGGTTAG
>**VvCIPK40**.V.vinifera v2.1|VIT_205s0020g00830.1
ATGGAGAGATTATACAATGAAGGGGGGGAGGGTGGTGGGATAAAGAGGGAGATTGCCACCTTGAAACTCCTCAAACATCCAAACGTGGTCAGATTACATGAGGTCTTGGCAAGCAAAACCAAGATTTACAT
GGTCCTTGAGTATGTGACTGGCGGCGAATTGTTTGACAGAATTGCATCCAATGGGAAACTTTCAGAAGCTGAAGGCAGGAAACTCTTCCAACAGTTGATTGATGCTGTGAGCTATTGCCACAACAAAGGTGTT
TTCCATAGGGATCTCAAGCTAGAGAATGTTCTTGTTGATTCAAAAGGAAACATAAAGATCTCTGATTTTGGCCTCAGTGCCTTACCACAGCATTTTAGGGATGATGGGTTGTTGCATACAACCTGTGGAAGTCC
AAACTACGTTGCACCAGAGATTCTTGCAAATAGAGGTTACGACGGTTCAACTTCTGACATTTGGTCATGTGGTGTCATCTTATACGTGATTCTTACAGGGTATCTCCCTTTTGATGATAGAAATCTTGCTGTTCT
CTATCAGAAGATTTTCAAGGGGGATGCTCAGATGCCCAAATGGCTGTCATCAGGTGCCAAGAACATTATAAAGAGAATTCTTGACCCCAAACCTGAAACCCGAATAACAATGGCAGGGATCAAAGCAGATGA
ATGGTTCAAGCAACAGTATACACCAGCTTATCCCGAAGAGGAAGAAGAAGATATTCATGTGGATGATGAAGCTTTCTCAATATATGAGCCAATGGAAGCAAAGAAGAGTCCAGGCTCACCCAACCTCATCAA
TGCCTTCCAGCTGATTGGAATGTCCTCATGCCTTGATCTCTCTGGTTTCTTTGAGAAAGAGGATGTGTCAGAGAGGAAAATCAGATTTACCTCAAATCATTGCCCAAAAGATTTACTTGAGAAGATTGAGGATA
TTGTCACAGAGATGGGATTTCGAGTAGAGAAGAAAAATGGAAGGCTTAAAGTGATGCAAGAGCATAAGTGTCAGAAAAGCTTTGGGAGTCTTTCCGTGGCAGCAGAGGTGTTTGAAATAAGCCCATCCTTGT
ATGTAGTTGAGTTGAGAAAATCTTATGGGGATTCTTCTGTTTATAGACAGTTGTGTAAAAAGCTATCAAGTGATCTGGGTGTTCCTACCAGCCAAGAGCTATTGAAAGTTTGA
>VvCIPK41-1.V.vinifera v2.1|VIT_218s0001g07980.1
ATGGTGGCGGTGCGGAAGGTTGGCAAGTATGAGGTGGGGAGGACGATCGGAGAAGGGACCTTCGCTAAGGTAAAGTTCGCGCAGAACACGGAGACCGGTGAGAGTGTGGCCATGAAAGTGCTCGATCGCA
GCACCATCATCAAGCACAAGATGACCGACCAGATTAAGAGGGAGATATCTATAATGAAGCTTGTTAGACATCCATATGTTGTTCGTCTTCATGAGGTTCTAGCAAGCCGTACAAAAATATATATAATCTTGGA
GTTCATCACAGGTGGCGAGTTGTTTGATAAAATAGTTCACCATGGCCGACTAAGTGAAAATGAATCTAGAAGTTACTTCCAACAGCTCATTGACGGTGTGGATTATTGCCACAGTAAGGGAGTCTACCACAGA
GACTTAAAGCCTGAAAACCTTCTACTAGATTCCCAAGGAAATCTAAAGATTTCAGATTTTGGTCTCAGTGCGTTGCCTGCGCCGGGAGTTAGCCTCCTTAAGACAACTTGTGGTACTCCTAACTATGTAGCACC
AGAGGTACTCAGTCACAAGGGTTATGATGGTGCTGTAGCAGATGTCTGGTCTTGTGGTGTCATCCTTTATGTTCTGATGGCTGGATATCTCCCGTTTGATGAGCTTGATTTAACCACTCTGTACAGTAAGATCG
AGAGAGCAGAATTTTCATGCCCATCCTGGTTTCCAGTGGGAGCAAAATCCTTGATTCATAGAATTTTAGACCCAAATCCTGAAACTCGCATTCGAATTGAACAGATCAGGAGCGATGAGTGGTTTAGAAAGG
GGTATGTTCCAGTCCGACTTCTTGAATATGAAGATGTTAACCTGGATGATGTAAACGCTGTGTTTGATGATCCTGAGGAACAACATGCTAATGATCAACATGGAAATGAGGACATGGGTCCTCTGATTCTTAA
TGCATTTGACCTAATAATTCTTTCTCAGGGCCTAAACCTTTCAGCTCTGTTTGAACGGGGAGAGGGCCCTGTAAAGCATCAAACACGTTTCGTTTCACAGAAACCACCAAGAGTTGTTCTATCAAGCATGGAA
GTTGTTGCACAATCCATGGGTTTCAAGACACATATTCGCAATTATAAGATGAGGGTGGAAGGCATTTCAGCCAATAAGATGGAACATTTCTCAGTCATGTTGGAAATTTTTGAAGTTGCTCCCACATTTATTAT
GGTAGACATTCAGAAAGCAGCTGGAGATGCTGGTGAATACCAGAAGTTTTACAAGAACTTCTGCAGCAATCTGGAGGATATCATCTGGAAACCACTCAATGAATCAAGCAAATCAAGGATCACCAAGACGA
AGAGTAAAAAGGGTTGA
>**VvCIPK41** -2.V.vinifera v2.1|VIT_218s0001g07980.2
ATGGTGGCGGTGCGGAAGGTTGGCAAGTATGAGGTGGGGAGGACGATCGGAGAAGGGACCTTCGCTAAGGTAAAGTTCGCGCAGAACACGGAGACCGGTGAGAGTGTGGCCATGAAAGTGCTCGATCGCA
GCACCATCATCAAGCACAAGATGACCGACCAGATTAAGAGGGAGATATCTATAATGAAGCTTGTTAGACATCCATATGTTGTTCGTCTTCATGAGGTTCTAGCAAGCCGTACAAAAATATATATAATCTTGGA
GTTCATCACAGGTGGCGAGTTGTTTGATAAAATAGTTCACCATGGCCGACTAAGTGAAAATGAATCTAGAAGTTACTTCCAACAGCTCATTGACGGTGTGGATTATTGCCACAGTAAGGGAGTCTACCACAGA
GACTTAAAGCCTGAAAACCTTCTACTAGATTCCCAAGGAAATCTAAAGATTTCAGATTTTGGTCTCAGTGCGTTGCCTGCGCCGGGAGTTAGCCTCCTTAAGACAACTTGTGGTACTCCTAACTATGTAGCACC
AGAGGTACTCAGTCACAAGGGTTATGATGGTGCTGTAGCAGATGTCTGGTCTTGTGGTGTCATCCTTTATGTTCTGATGGCTGGATATCTCCCGTTTGATGAGCTTGATTTAACCACTCTGTACAGTAAGATCG

AGAGAGCAGAATTTTCATGCCCATCCTGGTTTCCAGTGGGAGCAAAATCCTTGATTCATAGAATTTTAGACCCAAATCCTGAAACTCGCATTCGAATTGAACAGATCAGGAGCGATGAGTGGTTTAGAAAGG
GGTATGTTCCAGTCCGACTTCTTGAATATGAAGATGTTAACCTGGATGATGTAAACGCTGTGTTTGATGATCCTGAGGAACAACATGCTAATGATCAACATGGAAATGAGGACATGGGTCCTCTGATTCTTAA
TGCATTTGACCTAATAATTCTTTCTCAGGGCCTAAACCTTTCAGCTCTGTTTGAACGGGGAGAGTACTCCTGGGCTGTTAGGAATGTAAAAAATATCAGATCTGACCAATGA

>**VvCIPK41-**3.V.vinifera v2.1|VIT_218s0001g07980.3

ATGGTGGCGGTGCGGAAGGTTGGCAAGTATGAGGTGGGGAGGACGATCGGAGAAGGGACCTTCGCTAAGGTAAAGTTCGCGCAGAACACGGAGACCGGTGAGAGTGTGGCCATGAAAGTGCTCGATCGCA
GCACCATCATCAAGCACAAGATGACCGACCAGATTAAGAGGGAGATATCTATAATGAAGCTTGTTAGACATCCATATGTTGTTCGTCTTCATGAGGTTCTAGCAAGCCGTACAAAAATATATATAATCTTGGA
GTTCATCACAGGTGGCGAGTTGTTTGATAAAATAGTTCACCATGGCCGACTAAGTGAAAATGAATCTAGAAGTTACTTCCAACAGCTCATTGACGGTGTGGATTATTGCCACAGTAAGGGAGTCTACCACAGA
GACTTAAAGCCTGAAAACCTTCTACTAGATTCCCAAGGAAATCTAAAGATTTCAGATTTTGGTCTCAGTGCGTTGCCTGCGCCGGGAGTTAGCCTCCTTAAGACAACTTGTGGTACTCCTAACTATGTAGCACC
AGAGGTACTCAGTCACAAGGGTTATGATGGTGCTGTAGCAGATGTCTGGTCTTGTGGTGTCATCCTTTATGTTCTGATGGCTGGATATCTCCCGTTTGATGAGCTTGATTTAACCACTCTGTACAGTAAGATCG
AGAGAGCAGAATTTTCATGCCCATCCTGGTTTCCAGTGGGAGCAAAATCCTTGATTCATAGAATTTTAGACCCAAATCCTGAAACTCGCATTCGAATTGAACAGATCAGGAGCGATGAGTGGTTTAGAAAGG
GGTATGTTCCAGTCCGACTTCTTGAATATGAAGATGTTAACCTGGATGATGTAAACGCTGTGTTTGATGATCCTGAGGAACAACATGCTAATGATCAACATGGAAATGAGGACATGGGTCCTCTGATTCTTAA
TGCATTTGACCTAATAATTCTTTCTCAGGGCCTAAACCTTTCAGCTCTGTTTGAACGGGGAGAGGGCCCTGTAAAGCATCAAACACGTTTCGTTTCACAGAAACCACCAAGAGTTGTTCTATCAAGCATGGAA
GTTGTTGCACAATCCATGGGTTTCAAGACACATATTCGCAATTATAAGATGAGGGTGGAAGGCATTTCAGCCAATAAGATGGAACATTTCTCAGTCATGTTGGAAATTTTTGAAGTTGCTCCCACATTTATTAT
GGTAGACATTCAGAAAGCAGCTGGAGATGCTGGTGAATACCAGAAGGTTAGTTACTCAAAATTGTTCTCTTCACAACATATCTGA

>**VvCIPK41-**4.V.vinifera v2.1|VIT_218s0001g07980.4

ATGGTGGCGGTGCGGAAGGTTGGCAAGTATGAGGTGGGGAGGACGATCGGAGAAGGGACCTTCGCTAAGGTAAAGTTCGCGCAGAACACGGAGACCGGTGAGAGTGTGGCCATGAAAGTGCTCGATCGCA
GCACCATCATCAAGCACAAGATGACCGACCAGATTAAGAGGGAGATATCTATAATGAAGCTTGTTAGACATCCATATGTTGTTCGTCTTCATGAGGTTCTAGCAAGCCGTACAAAAATATATATAATCTTGGA
GTTCATCACAGGTGGCGAGTTGTTTGATAAAATAGTTCACCATGGCCGACTAAGTGAAAATGAATCTAGAAGTTACTTCCAACAGCTCATTGACGGTGTGGATTATTGCCACAGTAAGGGAGTCTACCACAGA
GACTTAAAGCCTGAAAACCTTCTACTAGATTCCCAAGGAAATCTAAAGATTTCAGATTTTGGTCTCAGTGCGTTGCCTGCGCCGGGAGTTAGCCTCCTTAAGACAACTTGTGGTACTCCTAACTATGTAGCACC
AGAGGTACTCAGTCACAAGGGTTATGATGGTGCTGTAGCAGATGTCTGGTCTTGTGGTGTCATCCTTTATGTTCTGATGGCTGGATATCTCCCGTTTGATGAGCTTGATTTAACCACTCTGTACAGTAAGATCG
AGAGAGCAGAATTTTCATGCCCATCCTGGTTTCCAGTGGGAGCAAAATCCTTGATTCATAGAATTTTAGACCCAAATCCTGAAACTCGCATTCGAATTGAACAGATCAGGAGCGATGAGTGGTTTAGAAAGG
GGTATGTTCCAGTCCGACTTCTTGAATATGAAGATGTTAACCTGGATGATGTAAACGCTGTGTTTGATGATCCTGAGGAACAACATGCTAATGATCAACATGGAAATGAGGACATGGGTCCTCTGATTCTTAA
TGCATTTGACCTAATAATTCTTTCTCAGGGCCTAAACCTTTCAGCTCTGTTTGAACGGGGAGAGGTAAGATTTACTTGTATTTTTATTGTATTCCACTCATATATATATATATATATATATATAGATGGATATATAT
ATATATATCTGACATCTGGTTTAGCAATCCTAACTAA

>**VvCIPK41-**5.V.vinifera v2.1|VIT_218s0001g07980.5

ATGGTGGCGGTGCGGAAGGTTGGCAAGTATGAGGTGGGGAGGACGATCGGAGAAGGGACCTTCGCTAAGGTAAAGTTCGCGCAGAACACGGAGACCGGTGAGAGTGTGGCCATGAAAGTGCTCGATCGCA
GCACCATCATCAAGCACAAGATGACCGACCAGATTAAGAGGGAGATATCTATAATGAAGCTTGTTAGACATCCATATGTTGTTCGTCTTCATGAGGTTCTAGCAAGCCGTACAAAAATATATATAATCTTGGA
GTTCATCACAGGTGGCGAGTTGTTTGATAAAATAGTTCACCATGGCCGACTAAGTGAAAATGAATCTAGAAGTTACTTCCAACAGCTCATTGACGGTGTGGATTATTGCCACAGTAAGGGAGTCTACCACAGA
GACTTAAAGCCTGAAAACCTTCTACTAGATTCCCAAGGAAATCTAAAGATTTCAGATTTTGGTCTCAGTGCGTTGCCTGCGCCGGGAGTTAGCCTCCTTAAGACAACTTGTGGTACTCCTAACTATGTAGCACC
AGAGGTACTCAGTCACAAGGGTTATGATGGTGCTGTAGCAGATGTCTGGTCTTGTGGTGTCATCCTTTATGTTCTGATGGCTGGATATCTCCCGTTTGATGAGCTTGATTTAACCACTCTGTACAGTAAGATCG
AGAGAGCAGAATTTTCATGCCCATCCTGGTTTCCAGTGGGAGCAAAATCCTTGATTCATAGAATTTTAGACCCAAATCCTGAAACTCGCATTCGAATTGAACAGATCAGGAGCGATGAGTGGTTTAGAAAGG
GGTATGTTCCAGTCCGACTTCTTGAATATGAAGATGTTAACCTGGATGATGTAAACGCTGTGTTTGATGATCCTGAGGTTGGAACAACATGCTAA

>**VvCIPK41-**6.V.vinifera v2.1|VIT_218s0001g07980.6

ATGGTGGCGGTGCGGAAGGTTGGCAAGTATGAGGTGGGGAGGACGATCGGAGAAGGGACCTTCGCTAAGGTAAAGTTCGCGCAGAACACGGAGACCGGTGAGAGTGTGGCCATGAAAGTGCTCGATCGCA
GCACCATCATCAAGCACAAGATGACCGACCAGATTAAGAGGGAGATATCTATAATGAAGCTTGTTAGACATCCATATGTTGTTCGTCTTCATGAGGTTCTAGCAAGCCGTACAAAAATATATATAATCTTGGA
GTTCATCACAGGTGGCGAGTTGTTTGATAAAATAGTTCACCATGGCCGACTAAGTGAAAATGAATCTAGAAGTTACTTCCAACAGCTCATTGACGGTGTGGATTATTGCCACAGTAAGGGAGTCTACCACAGA
GACTTAAAGCCTGAAAACCTTCTACTAGATTCCCAAGGAAATCTAAAGATTTCAGATTTTGGTCTCAGTGCGTTGCCTGCGCCGGGAGTTAGCCTCCTTAAGACAACTTGTGGTACTCCTAACTATGTAGCACC
AGAGGTACTCAGTCACAAGGGTTATGATGGTGCTGTAGCAGATGTCTGGTCTTGTGGTGTCATCCTTTATGTTCTGATGGCTGGATATCTCCCGTTTGATGAGCTTGATTTAACCACTCTGTACAGTAAGATCG
AGAGAGCAGAATTTTCATGCCCATCCTGGTTTCCAGTGGGAGCAAAATCCTTGATTCATAGAATTTTAGACCCAAATCCTGAAACTCGCATTCGAATTGAACAGATCAGGAGCGATGAGTGGTTTAGAAAGG
GGTATGTTCCAGTCCGACTTCTTGAATATGAAGATGTTAACCTGGATGATGTAAACGCTGTGTTTGATGATCCTGAGGTTGGTAAGGTTTCCAAATGTATTTTTTGTTTCTTAAATTTCCTGGTCTATGAAATAC
TGGGAAACAGTCTGTATTCATGCTTTTGCGTTTTATTGCCACTGTGA
